# Replication stress generates distinctive landscapes of DNA copy number alterations and chromosome scale losses

**DOI:** 10.1186/s13059-022-02781-0

**Published:** 2022-10-20

**Authors:** Nadeem Shaikh, Alice Mazzagatti, Simone De Angelis, Sarah C. Johnson, Bjorn Bakker, Diana C. J. Spierings, René Wardenaar, Eleni Maniati, Jun Wang, Michael A. Boemo, Floris Foijer, Sarah E. McClelland

**Affiliations:** 1 Barts Cancer Institute, Queen Mary University of London, London, EC1M 6BQ UK; 2 European Research Institute for the Biology of Ageing, University of Groningen, University Medical Center Groningen, A. Deusinglaan 1, Groningen, 9713 AV the Netherlands; 3 Current address: The Francis Crick Institute, 1 Midland Road, London, NW1 1AT UK; 4 Department of Pathology, University of Cambridge, Tennis Court Road, Cambridge, CB2 1QP UK

## Abstract

**Background:**

A major driver of cancer chromosomal instability is replication stress, the slowing or stalling of DNA replication. How replication stress and genomic instability are connected is not known. Aphidicolin-induced replication stress induces breakages at common fragile sites, but the exact causes of fragility are debated, and acute genomic consequences of replication stress are not fully explored.

**Results:**

We characterize DNA copy number alterations (CNAs) in single, diploid non-transformed cells, caused by one cell cycle in the presence of either aphidicolin or hydroxyurea. Multiple types of CNAs are generated, associated with different genomic regions and features, and observed copy number landscapes are distinct between aphidicolin and hydroxyurea-induced replication stress. Coupling cell type-specific analysis of CNAs to gene expression and single-cell replication timing analyses pinpointed the causative large genes of the most recurrent chromosome-scale CNAs in aphidicolin. These are clustered on chromosome 7 in RPE1 epithelial cells but chromosome 1 in BJ fibroblasts. Chromosome arm level CNAs also generate acentric lagging chromatin and micronuclei containing these chromosomes.

**Conclusions:**

Chromosomal instability driven by replication stress occurs via focal CNAs and chromosome arm scale changes, with the latter confined to a very small subset of chromosome regions, potentially heavily skewing cancer genome evolution. Different inducers of replication stress lead to distinctive CNA landscapes providing the opportunity to derive copy number signatures of specific replication stress mechanisms. Single-cell CNA analysis thus reveals the impact of replication stress on the genome, providing insights into the molecular mechanisms which fuel chromosomal instability in cancer.

**Supplementary Information:**

The online version contains supplementary material available at 10.1186/s13059-022-02781-0.

## Background

Most cancers exhibit chromosomal instability (CIN) that generates somatic copy number alterations, fuels tumor genome evolution, and is associated with poor prognosis. Replication stress drives chromosomal instability (CIN) in multiple cancer types [1, 2] and induced pluripotent stem cells [3] and can be driven by oncogenes [4–7], low nucleotide concentrations [8, 9], and difficult to replicate DNA sequences or structures [10, 11]. Stalled forks induced by replication stress trigger multiple responses (reviewed in Ref [10]). High levels of replication stress can activate cell cycle checkpoints and lead to senescence that can form a barrier to tumor initiation [12, 13]. Low levels can instead bypass DNA damage sensors ATR and Chk1 [14] allowing continued cell proliferation. It has also been shown that cells with under-replicated loci caused by replication stress can trigger a measure of last resort mitotic DNA synthesis (MiDAS) to promote replication at these regions prior to completion of mitosis [15–18]. Failure to use any or all of these pathways may result in double-strand breaks which could be translated into mitotic errors in the following cell division. Accordingly, it has been observed that replication stress can induce lagging chromatin, anaphase bridges, aneuploidy, and micronuclei formation [19, 20]. However, to date, the processes that convert replication stress intermediates into genomic alterations, whether chromosomal scale or sub-microscopic rearrangements, remain only partially characterized.

The consequences of replication stress on the genome have been mainly mapped by low-resolution cytogenetic analyses via the identification of chromosome gaps and breaks, occurring in genomic regions named common fragile sites (CFS). Replication stress-induced DNA copy number alterations (CNAs) have also been detected using array comparative genomic hybridization (aCGH) [21–24]. Binding sites of the Fancomi Anemia protein FANCD2 following replication stress were revealed using ChIP-Seq [25, 26], and MiDAS-Seq has mapped the genomic regions that frequently undergo mitotic DNA replication as a consequence of very late replication [27, 28]. Such approaches provide high positional resolution but are limited to the detection of those CNAs that survive long-term clonal outgrowth [21–23], or acute changes which appear frequently enough [24] to allow their detection using bulk population analysis. Replication stress-induced genomic alterations characterized so far may therefore represent only a minor fraction of the total effects of replication stress on the genome.

To understand the precise and acute changes to the genome upon replication stress, we analyzed CNAs induced in two diploid human cell types after one cell cycle under replication stress caused by low-dose aphidicolin, or hydroxyurea, using single-cell low-pass whole genome sequencing. This approach revealed multiple distinct classes of CNA, some of which were recurrent and clustered at characteristic genomic sites. A subset of large aphidicolin CNAs originate via chromosome breakage during mitosis. The resulting large chromosome fragments are mis-segregated at cell division, explaining chromosome segregation errors caused by replication stress, and are incorporated into micronuclei, a well-established intermediate of chromothripsis [29]. Surprisingly, this subset was confined to only a few breakpoint sites in RPE1 cells, resulting in the majority of acentric chromatin fragments and micronuclei comprising material from only three chromosomes. Large CNAs were generally associated with large or giant genes, and late replication timing. One highly recurrent fragile region of chromosome 7 in RPE1 cells correlated with RPE1-specific gene transcription of a nearby giant gene, AUTS2, while a similarly susceptible breakpoint region on chromosome 1 in BJ cells was driven by DAB1 expression. A second method to induce replication stress, hydroxyurea block and release, generated a pattern of CNAs that lacked a clear bias in position of chromosome arm scale CNAs, but were enriched at early replicating fragile sites (ERFS). Lastly, depletion of Mus81, a key endonuclease required for replication stress resolution, confirmed the key role of Mus81 in resolving replication intermediates and preventing chromosome arm scale CNAs in the presence of aphidicolin. Altogether, our study demonstrates that replication stress generates distinctive CNA spectra that likely reflect the mechanistic origin of genomic instability. Determining the types and locations of genomic aberrations caused by deregulation of specific replication and repair factors also provides the platform to further study their precise cellular functions in maintaining genomic stability.

## Results

### Treating cells with aphidicolin or hydroxyurea results in DNA damage and increased genomic instability

**Fig. 1:**
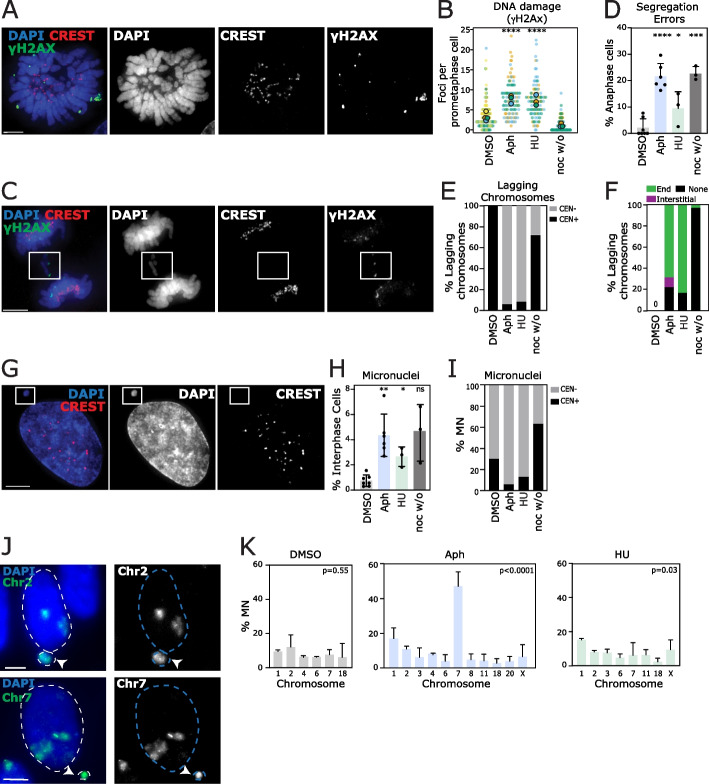
Replication stress induced by low-dose aphidicolin or hydroxyurea results in chromosome mis-segregation and micronuclei comprised of acentric chromatin. **A** Image of RPE1 prometaphase cell; DNA damage foci detected using γH2AX antibody. Scale bar in this and all subsequent microscopy images represents 5 μm. **B** Quantification of DNA damage in RPE1 prometaphase cells after indicated treatments; “noc w/o” indicates a nocodazole washout and release (see “Methods”). Statistical test was an unpaired *t*-test, comparing DMSO control to each individual condition. Data from at least three experiments, each in a separate color with mean for each experiment represented by large circle; *n*=94–147 total cells per treatment. **C** Immunofluorescence image of RPE1 anaphase cell with acentric lagging chromosome; centromeric proteins stained with CREST, DNA damage foci detected by γH2Ax staining. **D** Segregation error rates in RPE1 anaphase cells after indicated treatments (summary of three to seven experiments; *n*=105–313 cells, respectively; combined data from immunofluorescent and FISH analysis (see Figure S2f)); statistical test was an unpaired *t*-test. Each dot indicates mean of individual experiment. Error bars here and in all other figures indicate standard deviation. **E** Centromeric status of lagging chromosomes in RPE1 cells (as determined by CREST staining) after indicated treatments (*n*=2, 46, 7, or 37 lagging chromosomes, respectively, taken from at least three experiments). **F** Percentage of lagging chromosomes in RPE1 anaphase cells with DNA damage detectable on chromosome ends or within chromosome mass (*n*=0, 35, 12, and 37 lagging chromosomes scored in DMSO, aphidicolin, hydroxyurea, or nocodazole washout treatment, summary of three experiments). **G** Representative image of RPE1 cell with an acentric micronucleus. CREST antibody was used to stain for presence of centromeric proteins. **H** Quantification of micronuclei rates in RPE1 cells after indicated treatments (*n*=658–2150 cells respectively, taken from three to seven experiments) (see also Figure S2f for FISH staining). **I** Centromere status of micronuclei in RPE1 cells after indicated treatments (*n*=86–122 micronuclei per condition from three experiments), as determined by CREST staining (see also figure S2f). **J** Representative images of RPE1 cells treated with specific chromosome FISH probes to identify chromosomal identity of micronuclei. **K** Quantification of frequency with which indicated chromosomes were detected in micronuclei in RPE1 cells (summary of at least three experiments per chromosome tested, 50–100 micronuclei scored per chromosome per experiment). Statistical test was a one-way ANOVA

To study the effects of replication stress, we first used a diploid, telomerase-immortalized human cell line, retinal pigment epithelial (RPE1-hTERT, hereafter RPE1) cells. We chose two methods to induce replication stress (see Additional file 1: Fig S1a for workflow). One consisted of a continuous low dose of the DNA polymerase inhibitor, aphidicolin, for 24 h. The other method involved blocking cells in S-phase with hydroxyurea (HU) for 16 h, followed by release for 12–18 h. We used a variety of approaches to verify that both treatments were affecting cells in the expected manner (Additional file 1: Fig S2a-d). Immunofluorescent staining was then used to verify the presence of replication stress-induced defects. Aphidicolin and HU treatments induced elevated γH2AX foci in prometaphase cells, and increased rates of chromosome segregation errors and RPA-coated ultrafine bridges in anaphase cells (Fig. 1a–d; Additional file 1: Fig S2e). The majority of lagging chromatin fragments lacked a centromere (verified by both immunofluorescent staining for kinetochore proteins using CREST antibodies, or by fluorescent in situ hybridization (FISH) with pan-centromeric probes (Fig. 1c, e; Additional file 1: Fig S2f-h)). These fragments also frequently contained a focus of DNA damage at one, or both ends, as marked using antibodies to γH2AX (Fig. 1f). Alongside the increased segregation error rate, we observed a concomitant increase in micronuclei (MN) in interphase cells, which were also frequently acentric (Fig. 1g–I; Additional file 1: Fig S2f,h). For comparison, we also analyzed cells after a nocodazole washout to induce mitotic chromosome segregation errors due to improper microtubule attachments [30]. This treatment induced chromosome segregation errors and micronuclei that were more frequently centromere-positive and did not induce UFBs or γH2AX foci (Fig. 1a–I; Additional file 1: Fig S2e). Having established conditions for inducing replication stress by two different methods, we next moved to analysis of subsequent genomic alterations.

### Aphidicolin and hydroxyurea generate different patterns of chromosomes lost as micronuclei

We reasoned that analyzing the content of MN induced by replication stress could give insights into the genomic locations prone to genomic instability, in line with a previous study showing that expression of CFSs results in preferential loss of highly fragile genomic loci into micronuclei [17]. We used chromosome paints to identity the chromosomal material encapsulated within micronuclei arising after aphidicolin or HU treatment (Fig. 1j). In DMSO-treated cells, which harbor a low but analyzable proportion of micronuclei, there was no apparent bias between the chromosomes tested. By contrast, treatment with aphidicolin caused a significant bias towards chromosome 7 (nearly 45% of MN) and chromosome 1 (15%). By contrast, HU treatment showed only a mild (and non-significant) bias towards loss of chromosome 1 (Fig. 1k).

### Single-cell sequencing demonstrates that aphidicolin and hydroxyurea generate unique patterns of CNAs

**Fig. 2:**
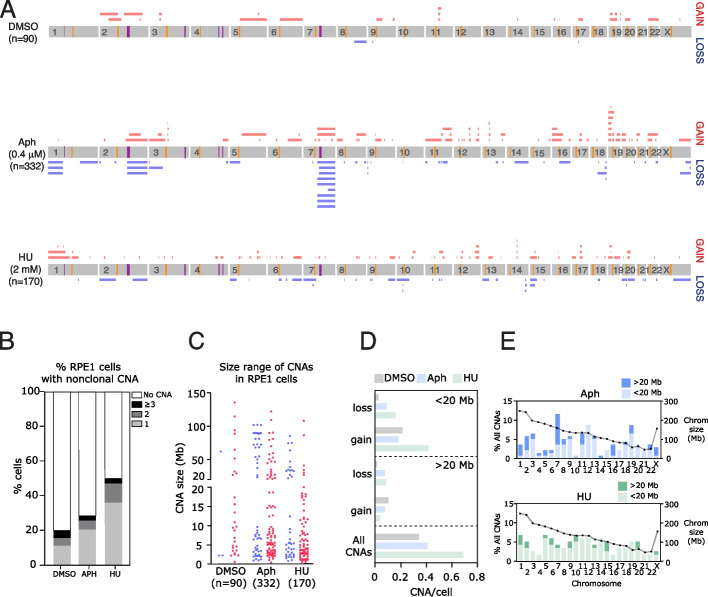
Genomic features of aCNAs detected by single-cell whole genome sequencing. **A** Diagrams summarizing all RPE1 CNAs induced by indicated treatments, after removing clonal events (see “Methods”). Yellow lines indicate location of centromeres, purple lines represent RPE1-specific CFS locations. **B** Frequency of CNAs occurring in RPE1 cells after treatments as indicated (*n* = 90, 332, or 170 cells, respectively). **C** Distribution of CNAs divided by size and by gains versus losses in RPE1 (31 CNAs identified in DMSO, 136 in aphidicolin, 118 in HU). **D** Rate of CNA classes per cell (small CNAs defined as less than 20 Mb; large defined as 20 Mb or larger). **E** Frequencies of CNAs (left *y*-axis) across each chromosome after aphidicolin or HU treatment as indicated, divided into large (>20 Mb) and small (< 20 Mb) CNAs. Dotted line indicates size of each chromosome (right *y*-axis)

We reasoned that, in addition to large genomic alterations visible as mis-segregating chromatin in mitosis, smaller genomic alterations may also be acutely induced by replication stress that may not have been detected with previous approaches. We therefore performed low-pass single-cell whole genome sequencing of G1 cells after replication stress induction, to detect whole and partial chromosome copy number changes [30–32] (Additional file 1: Fig S3a; Additional file 2). Since we were interested in the genomic alterations caused by acute replication stress induction before any selective pressure could shape the landscape of observed CNAs, we isolated G1 cells after 24 h of aphidicolin treatment (the majority of which had undergone DNA replication in the presence of aphidicolin (Additional file 1: Fig S2c,d)) or 18 h after release from HU (Additional file 1: Fig S2a,b). In both treatments, cells would have passed through a faulty S-phase, G2, and error-prone mitosis before their isolation in G1. Single-cell genome sequencing detected several classes of CNA, including a known sub-clonal trisomy of chromosome 12 and a clonal 10q amplification in RPE1 cells (Additional file 1: Fig S3b). To visualize the landscape of replication stress-induced CNAs, we discarded clonal and sub-clonal copy number alterations, and other CNAs that occurred in or near centromeric or telomeric regions that were potentially mapping artifacts (see Additional file 1: Figs S3, S4 and Methods), and collated CNA events from 332 single RPE1 cells treated with aphidicolin (Fig. 2a) and 170 cells treated with HU. Both aphidicolin and HU treatments led to an elevated rate of CNAs, with 29 and 50% cells exhibiting at least one aphidicolin-induced (hereafter “aCNA”) or hydroxyurea-induced (hCNA) copy number alteration (Fig. 2b). We noticed that aCNAs tended to fall into two size classes; focal amplifications or deletions between 1 and 20 Mb (small gains/losses), or much larger amplifications or deletions (>20 Mb, large gains/losses) that extended from a single breakpoint to the end of the chromosome arm (“large terminal CNAs”; Fig. 2c). Intriguingly, small and large aCNAs tended to occur at different genomic regions and were differently distributed across the genome (Fig. 2a). We hypothesized that different mechanisms may operate to drive these classes of lesion. Twenty percent of DMSO-treated cells also exhibited CNAs (particularly small gains). In an attempt to reduce background CNAs, we single-cell cloned RPE1 cells, before single-cell sequencing. However, this did not reduce the background rate of CNAs (Additional file 1: Fig S4f,g) suggesting the DMSO CNA rate is either an artifact of the sequencing process, or alternatively accurately reflects an ongoing rate of small CNA accumulation. Overall, and taking into account the number of cells sequenced for each condition, we calculated that both aphidicolin and hydroxyurea treatments generated increases in small losses (3.8 and 7.1-fold increase) and large losses (6.5 and 7.4-fold) when compared to DMSO controls. HU also caused a 2-fold increase in small gains (Fig. 2d). We wondered if there were particular hotspots for replication stress-induced CNAs and plotted the distribution of CNAs across all chromosomes (Fig. 2e). We noted that several chromosomes accounted for the majority of aCNAs (in particular chromosome 7), whereas the hCNAs were more evenly distributed. We wondered if this could be explained by chromosome size, but there was no indication that larger chromosomes harbored more CNAs than smaller ones (Fig. 2e).

### The distribution of aphidicolin CNAs across the genome differs between RPE1 and BJ cells

**Fig. 3:**
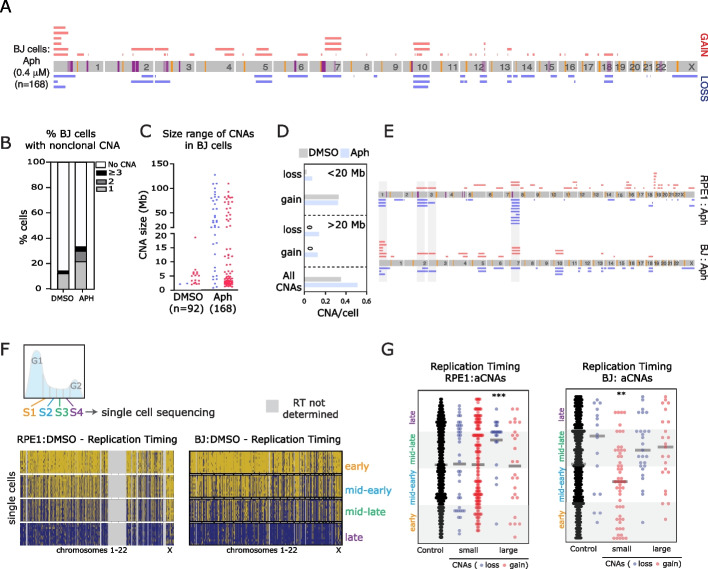
Transcription of giant genes can underlie cell type-dependent susceptibility to large aCNAs. **A** Diagram summarizing all BJ aCNAs taken from 168 cells, after removing clonal events (see “Methods”). Yellow lines show centromeres, purple lines indicate positions of BJ-specific CFS. **B** Frequency of CNAs occurring in BJ cells after DMSO or aphidicolin as indicated (*n* = 92 and 168 cells respectively). **C** Range of sizes of CNAs divided into loss and gain in BJ cells (32 CNAs identified in DMSO, 85 in aphidicolin). **D** Frequency of small or large CNAs for each chromosome in BJ cells after aphidicolin treatment. **E** Map of large CNAs in RPE1 and BJ cells after aphidicolin. **F** Upper panel; schematic indicating S-phase fractions isolated using FACS for single-cell replication timing. Lower panel: single-cell replication timing analyses for RPE1 and BJ cells as indicated from each S-phase fraction. Dark blue indicates replicated genomic regions. **G** Replication timing factor (see “Methods”) was plotted for each aCNA in RPE1 and BJ cells, and compared to random control regions (470 in silico randomly generated 2 Mb windows, see “Methods”). Statistical tests compare all aCNA classes to in silico control CNAs using a one-way ANOVA Kruskal-Wallis test with post hoc Dunn’s correction

Common fragile sites vary in their expression frequencies between different cell types [33–35]. The precise reasons for variation in the frequency of specific fragile sites between different cell types are not clear but likely depend on cell type-specific replication timing and gene expression programs. We therefore repeated our aphidicolin treatment and single-cell sequencing analysis on another diploid human cell line derived from foreskin fibroblasts (BJ-hTERT, hereafter “BJ”) to see whether aCNAs differed between the two cell lines. As with RPE1 cells, aphidicolin treatment induced DNA damage, segregation errors (which were biased towards acentric fragments often exhibiting DNA damage on one or both ends), and an increased rate of micronuclei in BJ cells (Additional file 1: Fig S5a-i). We single-cell sequenced 92 DMSO and 168 aphidicolin-treated G1 BJ cells (Additional file 1: Fig S5j). As before, we removed CNAs that were recurrent across multiple cells and likely represented sub-clonal or clonal events (Fig. 3a). Aphidicolin treatment increased the rate of cells with one or more non-clonal CNAs from 14 to 33% (Fig. 3b). Small losses, and large losses and gains all demonstrated increased CNA rates per cell compared to DMSO-treated BJ cells (Fig. 3c, d). We noted some common affected genomic regions between the two cell lines (on chromosomes 1, 2, and 7). Overall however, this analysis revealed a different distribution of large terminal aCNAs between BJ and RPE1 cells (Fig. 3e; Additional file 1: Fig S5k), suggesting that cell type-specific features could underlie these different distributions.

### Genomic regions of large (arm-scale) losses tend to replicate late in S-phase

Genome replication is an ordered process with most regions of the genome consistently being replicated at a specific time window during S-phase [36, 37]. Fragile sites have been associated with both late- (common fragile sites [21, 38–40]) and early-replicated (early-replicated fragile sites (ERFS) [41]) genomic regions. Replication timing programs can vary between cell types, and therefore, we performed single-cell sequencing-based replication timing analysis in a similar manner to recent studies [42, 43] on RPE1 and BJ cells, in order to precisely analyze the relationship between CNA position and specific replication timing. We isolated cells from four separate S-phase fractions (Fig. 3f) before single-cell sequencing to determine copy number profiles (Fig. 3f and Additional file 3). This provides, to our knowledge, the highest resolution replication timing profile of human cells using single-cell sequencing to date. Replication timing profiles from single cells were similar to replication timing profiles derived from sequencing of bulk populations (Additional file 1: Fig S6a). This provided the opportunity to define the replication timing based on the proportion of cells that had replicated a given 1 Mb genomic region as a sum score across the four quartiles of S-phase (“Replication timing factor,” see Methods). If that region was frequently replicated early in most or all single cells, then we attributed a high score; low scores denoted regions that were frequently replicated late in S-phase (or not replicated at all). In this, and all following analyses, we analyzed all CNA categories for completeness although only a subset of these were clearly elevated following aphidicolin treatment in RPE1 or BJ cells (see above). In RPE1 cells, breakpoints of large terminal losses displayed replication timing that was significantly later than a set of randomly placed 1 Mb CNAs that serve as an in silico control (see “Methods”; Fig. 3g). In BJ cells, breakpoints of large terminal losses also had a tendency towards mid-late or late replication, although this was not significant (*p*=0.06); small gains were earlier replicating, but since the rates of this CNA class in both DMSO and aphidicolin were similar (Fig. 3d), the significance of this finding remains unclear. Other CNA types did not show significantly different timing.

Recent studies suggested that genomic regions containing common fragile sites were not necessarily replicated late under normal conditions, but instead were associated with regions of early/mid S-phase replication that were shifted to later replication under replication stress [34, 44]. We therefore repeated single-cell sequencing-based replication timing analysis in cells treated with aphidicolin (Additional file 1: Fig S6b). Overall, there was a similar enrichment of large losses in late replicated regions as seen for DMSO replication timing, although this was now only borderline significant (*p*=0.059; Additional file 1: Fig S6c). We also tested whether there was an enrichment of aCNAs in regions that displayed a shift in replication timing (ΔRT) to earlier or later in S-phase, in response to aphidicolin. Previous studies showed 1.5–4% of the genome is subject to aphidicolin-induced shifts in replication timing [34, 44, 45]. We therefore analyzed 1 Mb bins that fell within the 5% extreme of the normal distribution of ΔRT (2.5% at each end of the distribution representing shifts towards much later, or much earlier timing in aphidicolin when compared with DMSO (Additional file 1: Fig S6d)). This analysis revealed an association of breakpoints of large deletions with regions that shifted to much later replication timing, but this was driven entirely by the CNAs clustered within a small region on chromosome 7. Aside from that region, the enrichment for aCNAs in ΔRT regions was in general no stronger than for our random control regions (Additional file 1: Fig S6e), suggesting that late, rather than altered, timing was the major genome-wide factor relating to replication timing contributing to aCNAs in RPE1 and BJ cells.

### CNA breakpoints are not associated with high gene transcription

**Fig. 4:**
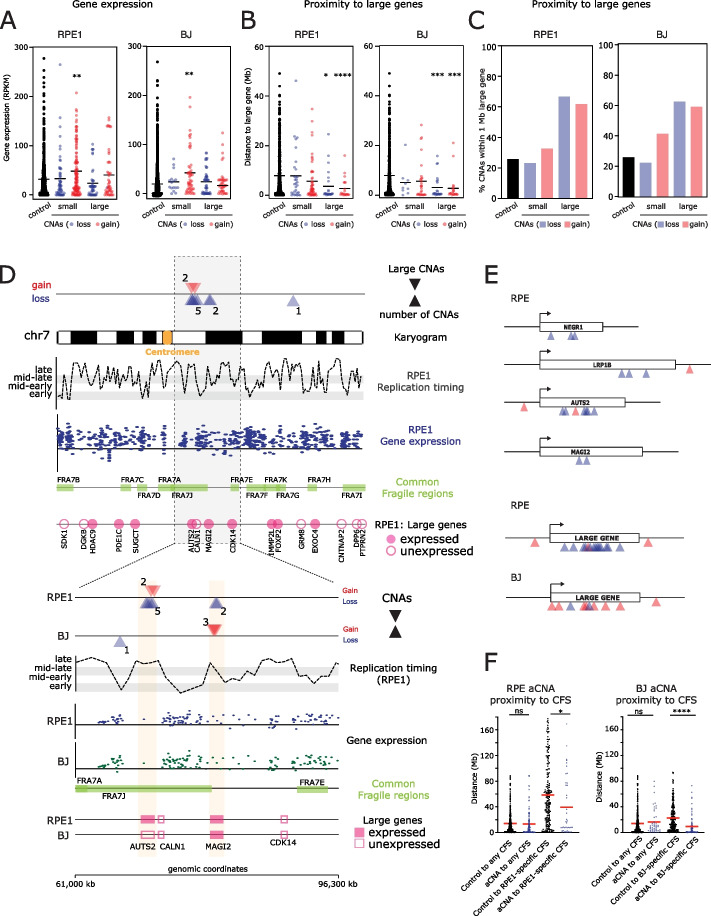
Large gene transcription underlies large, cell type-specific, recurrent aCNAs. **A** Summed gene expression within a 2 Mb window around each aCNA breakpoint, separated into CNA classes as indicated for RPE1 and BJ cells. **B** Distances from individual aCNAs to the nearest large or giant gene. Control represents 432 randomly generated genomic coordinates. **C** Proportion of CNAs with a breakpoint that falls within 1 Mb of large/giant gene. **D** Schematic of chromosome 7, with position of large (>20 Mb) CNAs found in RPE1 and BJ cells, with replication timing profile, gene expression (each dot indicates location and expression level (RPKM values, mean of three replicates) of an individual gene), location of human chromosome 7 CFSs compiled from the literature, location and expression status of nearby large genes. Zoom of selected portion indicates genomic characteristics and CNA positions in BJ and RPE1 cells. **E** Schematic indicating positions of aCNAs relative to large genes (with specific examples in RPE1 and summary for all large genes for two cell lines). **F** Proximity of random control sites or aCNAs to either all human CFS or cell line-specific CFS (RPE1-specific CFS taken from [33], BJ-specific CFS taken from [34]). Statistical tests compare all aCNA classes to in silico control CNAs using a one-way ANOVA Kruskal-Wallis test with post hoc Dunn’s correction

Previous studies have suggested a role for large genic transcription units in triggering hotspots of CNAs [21]. We therefore performed RNA sequencing (Additional file 3) to determine the abundance of all gene transcripts genome-wide in RPE1 and BJ cells treated with DMSO or aphidicolin. In line with previous studies [21, 34], aphidicolin treatment did not obviously impact gene expression globally (Additional file 1: Fig S7a). To ask whether high local rates of transcription could be a trigger for CNA formation, we analyzed the summed gene expression within a 2-Mb window centered around breakpoints for all four CNA classes and compared it to gene expression within windows at random in silico placed breakpoints as a control. We found that in both cell lines, only small gains were associated with a slightly higher level of transcription when compared with other types of CNA or with random genomic regions (Fig. 4a). However, this class of CNA was not increased compared to DMSO-treated cells (Figs. 2d and 3d) so the significance of this observation remains unclear.

### Large terminal CNAs occur close to large or giant genes

Large (>600 kb) or giant (>1 Mb) genes have been previously associated with fragility under replication stress [40, 46, 47] although the exact causes remain debated. We therefore analyzed the distance from aCNAs to the nearest large gene. For large terminal losses and gains in both in RPE and BJ cells, the distance to the nearest large gene was significantly lower compared to the in silico control regions (Fig. 4b). Moreover, the majority (59–66%) of large losses and gains were found to be in close proximity (<1 Mb) to a large or giant gene in RPE1 and BJ cells respectively (Fig. 4c). Taken together, these data show that breakpoints of large terminal aphidicolin-induced CNAs, in addition to being late replicated, are frequently in proximity to large or giant genes, aligning them with previously identified common fragile site features.

### Transcription of giant genes can underlie cell type-dependent susceptibility to large aCNAs

We noticed that the region of chromosome 7q that was highly prone to large terminal aCNAs in RPE1 cells was far less frequently altered in BJ cells, and the reverse was true for a specific region on chromosome 1 (more affected in BJ then RPE1 cells) (Fig. 3e). To determine the etiology of the highly recurrent CNAs in these regions and to understand why they were specific to the individual cell lines, we visualized the genomic features of these regions obtained from analyses above (Fig. 4d; Additional file 1: Fig S7b). It has been previously shown that proximity to large genes combined with late or inefficient replication predisposes to fragility in many CFSs studied [44]. Moreover, we had observed an enrichment of aCNAs in late replication timing regions (see above). As seen for large terminal CNAs in general (Fig. 4b,c), these recurrent large aCNA-associated fragile regions were often very close to large/giant genes. Namely, AUTS2 and MAGI2 are giant genes close to the RPE1-specific aCNAs on chromosome 7q (Fig. 4d). However, replication timing at large genes closest to the recurrent CNAs was similar between both cell types suggesting this was not a major factor in RPE1-specific fragility of these regions (Fig. 4d and data not shown). In both RPE1 and BJ cells, the breakpoints involved in the large terminal CNAs on chromosome 7 were found in a region containing three large genes: AUTS2, CALN1, and MAGI2. In particular, the RPE1-specific aCNAs fell in the proximity of AUTS2 and MAGI2, while the BJ-specific CNAs fell only near MAGI2 (Fig. 4d). From our RNA-seq data (see above), it emerged that AUTS2 and MAGI2 were both expressed in RPE1 cells, while in BJ cells only MAGI2 was expressed (Fig. 4d, Additional file 1: Fig S7c). In addition, for both cell lines, the aCNAs lacked any spatial correlation with large gene CALN1, which was unexpressed in both cell lines. This leads us to conclude that gene expression is a requirement for the fragility of these large gene-associated sites, in line with a previous study [21]. We then tested the relationship to large gene expression of the aCNAs on chromosome 1. We noted that aCNAs in BJ cells frequently occurred in close proximity to the large gene DAB1, which is expressed in BJ cells but not RPE1 (Additional file 1: Fig S7c). By contrast, we observed frequent large CNAs in both cell lines near large gene NEGR1, which was expressed in both cell lines (Additional file 1: Fig S7b,c). In all cases, expression of these large genes was not significantly altered in aphidicolin when compared to DMSO (Additional file 1: Fig S7a,c) suggesting the steady-state gene expression is sufficient to predispose to genomic alterations following aphidicolin treatment. These data suggest we are able to pinpoint the genes responsible for precipitating the most common large aCNAs in RPE1 and BJ cells (AUTS2 and MAGI2 on chromosome 7 and DAB1 and NEGR1 on chromosome 1). To further analyze the relationship between recurrent aCNAs and large genes, we plotted the position of large terminal aCNA breakpoints relative to their close-by or encompassing large/giant genes and their promoters. This revealed that large/giant gene-proximal aCNAs tended to originate within the central region of the genes, rather than the 5′ or 3′ ends specifically, in line with a previous study examining FANCD2 binding [25] (Fig. 4e). Overall, cell type-specific gene expression at large genes drives large terminal CNAs. These coincide with positions of cell type-specific fragile sites, but not common fragile sites in general (Fig. 4f; also see Figs. 2a, and 3a).

### Repetitive regions prone to forming secondary structures associate with small losses

A recent study [48] suggested that repeats composed of novel secondary structure-forming sequences are prone to breakage under ATR inhibition, raising the possibility that these secondary structure-forming sequences could predispose to replication stress-induced CNAs. Using motif enrichment analysis software (“Methods”), we analyzed the distribution of repeat clusters across the genome. We partitioned the genome into 1-Mb bins and ranked these in order of concentration of repeat-forming sequences. We noted that breakpoints of 75% of small aphidicolin losses were found in the top 50% of bins with the highest level of repeats (false discovery rate adjusted *p*=0.04, Chi-Square Goodness of Fit Test), suggesting secondary structure formation could contribute to this class of aCNA.

### hCNAs differ in most features of aCNAs except proximity to early replicating fragile sites

**Fig. 5:**
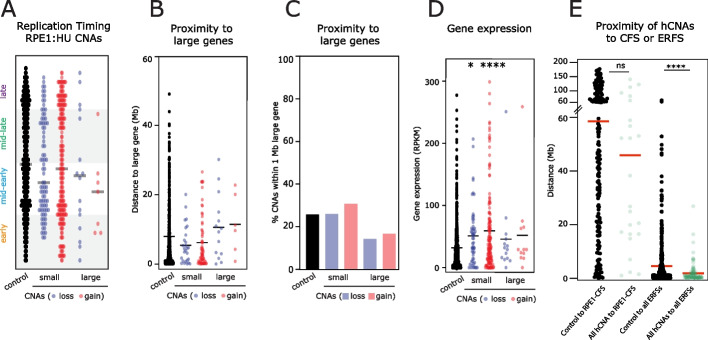
HU treatment generates a distinctive pattern of CNAs. **A** Replication timing of HU CNAs. **B** Proximity to large genes. **C** Proportion of hCNAs found within 1 Mb of a large gene. **D** Gene expression in 2 sMb windows around hCNA breakpoints. **E** Proximity of control sites or hCNAs to RPE1-specific CFS or all human ERFS. Statistical tests compare all hCNA classes to in silico control CNAs using a one-way ANOVA Kruskal-Wallis test with post hoc Dunn’s correction

Since the mechanisms of action of aphidicolin and HU are different, and patterns of micronuclei content (Fig. 1k) and CNA landscapes (Fig. 2) caused by HU differed from those of aCNAs, we wondered if this would be reflected in the features of genomic regions affected by copy number alterations in each condition. The same analyses were thus systematically conducted on the genomic regions affected by hCNAs detected by single-cell sequencing. In contrast to CNAs induced by aphidicolin treatment, we found no association for any of the hCNA classes with early or late replication timing, nor was there an increased proximity to large genes, nor secondary structure-forming repeat clusters (Fig. 5a–c). There was a small, but significant, increase in transcription levels near small hCNAs compared to the in silico control (Fig. 5d). Unlike aCNAs, there was no enrichment of hCNAs near RPE1-specific CFS loci (Fig. 5e). However, we did note that hCNAs were significantly closer to early replicating fragile sites (ERFS) (as defined by mapping regions of HU-induced RPA binding in lymphocytes [41]) (Fig. 5e). aCNAs also showed a similar association with ERFS (data not shown). Overall, CNAs caused by HU differ in several aspects from those caused by aphidicolin, and further examination of their features is likely to offer insights into mechanisms of fork restart.

### Depletion of Mus81 in RPE1 cells exacerbates the bias towards large aCNAs at recurrent sites in chromosomes 1, 2, and 7

**Fig. 6:**
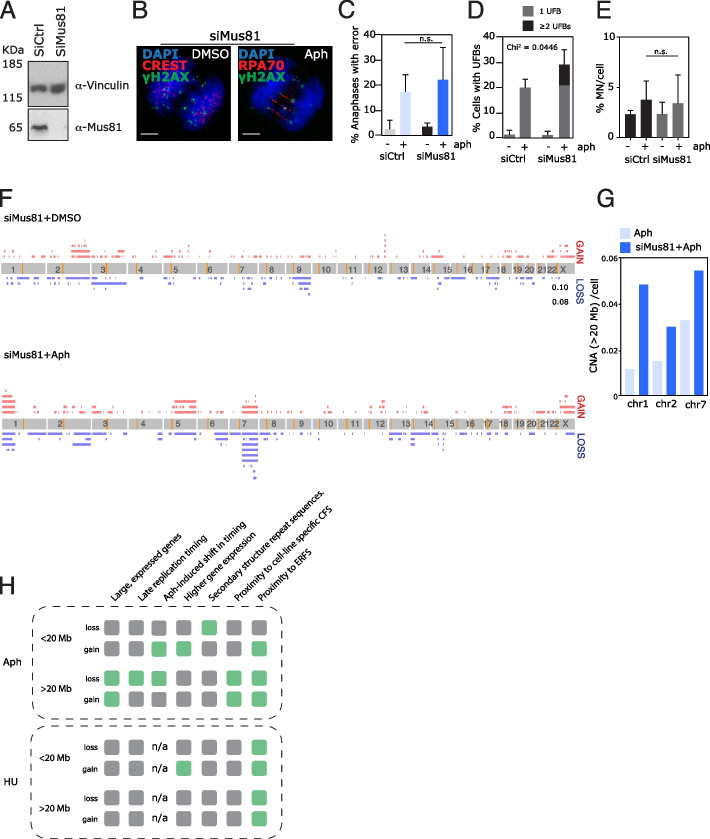
Depletion of Mus81 in the presence of aphidicolin exacerbates the bias towards specific sites of large aCNAs. **A** Western blot to indicate loss of Mus81 protein in RPE1 cells after siRNA for 48 h. Vinculin was used as a loading control. **B** Immunofluorescence image of RPE1 cell going through anaphase, with ultrafine bridge, as detected by replication protein A (RPA70). **C** Quantification of segregation error rates in RPE1 in combination treatments of siControl, siMus81 with either DMSO or aphidicolin. **D** Quantification of occurrence of ultrafine bridges in anaphase cells. **E** Quantification of rates of micronuclei from cells treated as indicated. **F** Summary of CNAs identified in RPE1 cells after siRNA depletion of Mus81, combined with either DMSO or aphidicolin. **G** Rate of large CNAs occurring on chromosomes 1, 2, and 7 in RPE1 cells (in aphidicolin, or in aphidicolin after depletion of Mus81). **H** Summary of genomic features linked to aCNAs and hCNAs identified in this study

We reasoned our single-cell sequencing approach could be used to gain insights into the mechanism of action of components of the cellular replication response pathways. Mus81 is an endonuclease responsible for cleaving replication intermediates and promoting mitotic DNA replication (MiDAS) [15, 16], and we wondered if the precise genomic regions rendered more fragile in the absence of Mus81 could be identified. We therefore depleted Mus81 from cells using siRNA and performed single-cell sequencing with or without aphidicolin treatment (Fig. 6a; Additional file 1: Fig S8a). Depletion of Mus81 alone did not cause an observable increase in DNA damage, segregation errors, ultrafine anaphase bridges, or micronuclei (Fig. 6b–e). In contrast to previous studies in other cell types [49], siMus81 depletion alone thus did not appear to cause notable loss of genome integrity in RPE1 cells, potentially due to cell type differences or transformation status. When combined with aphidicolin treatment, however, Mus81 depletion led to increased DNA damage and UFBs, in accordance with Mus81’s role in protecting against aphidicolin-induced genome instability [50], and cells with more than one UFB became more prevalent (Fig. 6d). Although a generally increased background rate of CNAs after siRNA treatments prevented detailed analysis of CNAs between the four conditions (aphidicolin, siControl, siMus81, and siMus81 + aphidicolin) (Additional file 1: Fig S8b), we noted that siMus81 combined with aphidicolin treatment resulted in an exaggerated bias towards large CNAs. Specifically, the rates of large CNAs at aphidicolin-sensitive regions on chromosomes 1, 2, and 7 were increased by 4, 2, and 1.6 fold respectively (Fig. 6f,g). Overall, these data reveal that Mus81 plays an important role in maintaining genomic stability at a subset of genomic regions associated with late replication timing and expression of nearby large genes.

## Discussion

Here, motivated by a desire to understand the mechanisms that convert replication stress to genome evolution during cancer, we sought to comprehensively assess the impact that replication stress has on the genome. Using a single-cell sequencing approach, we were able to detect CNAs caused by replication stress within a single-cell cycle and in the absence of any selective pressure, providing a comprehensive and unbiased analysis of genomic instability caused by two distinct classes of replication stress. We also analyzed DNA replication timing at the single-cell level, and bulk RNA sequencing to analyze gene expression in the same cell types, providing a complete picture of the factors involved in precipitating CNAs during replication stress from two different cell types. Previous studies tended to focus on a small number of recurrent fragile sites to understand molecular mechanisms operating to drive fragility under replication stress, potentially underlying some of the apparent conflicting views on the causes of fragility. Major advantages of our single-cell sequencing approach include the ability to identify all fragile regions including those that convert to large chromosomal breaks and gaps, and also those that result in small copy number alterations at a resolution of 0.5–1.6 Mb.

### What is special about recurrent sites of large terminal CNAs?

Large and giant genes have been proposed to cause fragility under replication stress due to a number of mechanisms including late [21, 38–40] or delayed replication timing [34, 44], low origin density [51], replication-transcription collisions during S-phase [5, 21, 52], and gene expression in G1 that removes licensing complexes and reduces origin density [44, 53, 54]. Large losses induced by aphidicolin were enriched in regions of late replication timing, with one recurrent region (AUTS2) also subject to an aphidicolin-induced shift from mid-late, to late replication. In addition, an interesting association was noted with proximity to expressed large or giant genes, which explained differences in aCNA landscapes between different cell types. In the case of the most highly recurrent sites of cell type-specific aCNAs, this was likely due to the RPE1-specific gene expression of the giant gene AUTS2, and the BJ-specific expression of DAB1. Although the association between large genes and fragile regions is well established, pinpointing the exact gene(s) and mechanisms responsible for fragility in each fragile site (often spanning very large regions) is challenging with cytogenetic techniques, requiring painstaking generation of region-specific FISH probes [33, 34]. Single-cell genomic sequencing thus provides a complementary approach to examine causes of replication stress-induced fragility at higher resolution in a genome-wide manner. We note that there are numerous expressed, late replicating, large genes residing within previously identified common fragile sites that do not precipitate CNAs following aphidicolin treatment (for example see chromosome 7 in Fig. 3b and chromosome 1 in Additional file 1: Fig S5). Only 10% of these locations were associated with a CNA, suggesting additional factors may also contribute to the extreme bias towards fragility of the recurrent large terminal CNAs observed in this study that we have not yet been able to identify. Furthermore, causative factors could vary between specific fragile sites, highlighting the importance of assessing causative features across numerous fragile sites in a genome-wide manner.

### Different replication stressors impact the genome in distinctive ways that can inform mechanisms of replication stress

Aphidicolin and HU are often used interchangeably to study cellular responses to replication stress. We showed however that each of these treatments generated distinctive CNA landscapes, with CNAs associated with different features (see schematic in Fig. 6h). Aphidicolin induced small losses and arm-scale CNAs, with the breakpoints of the latter associated with late replication timing and proximity to large expressed genes, giving rise to cell type-specific large genomic alteration patterns. Hydroxyurea-induced replication stress generated a pattern of genomic alterations quite distinct to that of aphidicolin. There was little enrichment for any particular chromosome, nor was there any detectable association with late replication timing, or proximity to large expressed genes. These data are seemingly in contrast to a previous study that found that CNAs generated by aphidicolin, HU, and irradiation tended to cluster in the same hotspots [21]. These differences may be due to the fact that we analyzed the acute short-term changes induced by replication stress in contrast to CNAs that were detectable after clonal growth and were thus subject to evolutionary selection and potential secondary genomic events during continued proliferation. In addition, it is possible that the cell type (RPE1), or specific HU block and release schedule we used could underlie differences between the hCNAs identified herein and the previous study. Depletion of Mus81 from cells served to exacerbate the rate of large CNAs formed by aphidicolin, further corroborating previous data relating to the role of Mus81 in the resolution of replication intermediates [49]. This finding reveals the utility of our approach to gain additional insights into mechanisms involved in cellular replication stress response pathways.

In addition to our study, other mechanism-specific CNA profiles are emerging: Array CGH and single-cell sequencing of tumors formed in mouse models of deregulated Mre11 (DNA double-strand break repair factor) and the DNA replication licensing factor MCM2 revealed distinctive CNA landscapes biased towards small deletions, although these were necessarily the product of both mutation and selection during tumor evolution [55, 56]. Single-cell sequencing of p53 null RPE1 cells following Cyclin E1 or CDC25 overexpression also revealed distinctive CNA landscapes, characterized by focal CNAs and large terminal CNAs respectively [57]. Discovering the precise mechanisms causing CNAs caused by aphidicolin and HU, in addition to those caused by other replication stressors, will likely uncover new insights into the mechanisms of specific components of the replication stress response. In turn, such analyses will be important to discover the etiology of disease-associated copy number and structural variations currently being uncovered at rapid rates from genome sequencing studies [58, 59].

### Replication stress could act to drive non-random CIN early in disease

Our data shows replication stress generates small CNAs that we identified from G1 cells following faulty S-phase, and thus appear not to activate cellular checkpoints, suggesting replication stress could act as a “stealth” CIN mechanism in the presence of functional cellular checkpoints and challenging the view that overcoming DNA damage-induced cellular senescence is a necessary step for CIN and tumor formation [13]. Further analysis of the sizes and locations of CNAs that do, or do not, elicit cell cycle arrest and senescence will be an important route to determining the potential for replication stress to act as an early driver of CIN and tumorigenesis without the need to overcome normal cellular checkpoints. Moreover, the observation that in RPE1 cells locations of large, chromosome arm scale events were essentially confined to three discrete loci affecting only chromosomes 1, 2, and 7 suggests that replication stress has the potential to heavily shape the evolution of tumor genomes in a non-random manner, similarly to our previous observations of non-random chromosome segregation caused by mitotic defects [30, 60].

## Conclusions

Single-cell sequencing identified aphidicolin-induced patterns of large gains and losses in genomic regions prone to physical fragility and in close proximity to large, late-replicating, expressed genes, as well as small losses in regions of repetitive sequences that tend to form secondary structures, in a cell type-specific manner. By contrast, hydroxyurea generated a different pattern of small and large gains and losses, which correlated only with ERFS.

## Methods

### Cell culture and RNAi

All cell lines were maintained at 37°C with 5% CO_2_. hTERT-RPE-1 cells were cultured in DMEM Nutrient Mixture F12 Ham (Sigma), and BJ cells in DMEM high glucose (Sigma). Media for both was supplemented with 10% FBS and 100 U Penicillin/Streptomycin. RPE1 and BJ cells were subjected to STR profiling to verify their identity using the cell line authentication service from Public Health England. Replication stress was induced using 0.4μM Aphidicolin (Aph) (Sigma) for 24 h (at doses that have been shown to induce CFS expression in RPE1 cells [33]), or 2mM hydroxyurea (HU) (Sigma) for 16 h, followed by washout for either 12 or 18 h. Nocodazole washout (noc w/o) involved adding 100ng/ml nocodazole for 4 h followed by drug washout for 1 h or for 16 h. RNAi was achieved by transfection of cells for 48 h with 20 nM small interfering RNA (siControl [D-001210-02] and siMus81 [CAGCCCUGGUGGAUCGAUAUU], Dharmacon) using Lipofectamine RNAiMAX (Invitrogen) and Optimem (Gibco). Medium was replaced with fresh media 24 h after addition of siRNA, and either DMSO or aphidicolin added 48 h after addition of RNAi for a further 24 h.

### Western blotting

Cell lysates were prepared using lysis buffer (20mM Tris-HCl, pH 7.5, 1% Triton X-100, 150mM NaCl, 5mM EDTA, 50mM NaF, 1mM PMSF, protease inhibitors (Roche)). Immunoblots were probed using antibodies against Mus81 (M1445, Sigma Aldrich) or Vinculin (Cambridge Bioscience 66305) and developed by exposing to X-ray film (Kodak) after using horseradish peroxidase-conjugated secondary antibodies (Santa Cruz).

### Immunofluorescence (IF)

Cells grown on glass slides or coverslips were fixed with PTEMF (0.2% Triton X-100, 0.02 M PIPES (pH 6.8), 0.01 M EGTA, 1 mM MgCl_2_, 4% formaldehyde). After blocking with 3% BSA, cells were incubated with primary antibodies according to suppliers’ instructions: CREST (Antibodies Incorporated, 15-234-0001), γH2aX (Millipore, 05-636), RPA70 (Abcam, ab79398). Secondary antibodies used were goat anti-mouse AlexaFluor 488 (A11017, Invitrogen), goat anti-rabbit AF594, AF488 (A11012, A11008, Invitrogen), and goat anti-human AF647 (109-606-088-JIR, Stratech or A21445, Invitrogen). DNA was stained with DAPI (Roche) and coverslips mounted in Vectashield (Vector H-1000, Vector Laboratories). EdU incorporation and staining was achieved using the Click-It kit (Life Technologies), following the manufacturer’s instructions.

### Fluorescence in situ hybridization (FISH)

Cells were grown on glass slides, fixed in methanol/acetic acid, then put through an ethanol dehydration series. Pan-centromeric probe (Cambio) was denatured at 85°C for 10 min then applied to slides, which were then incubated in a humidified chamber overnight at 37 °C. The following day, slides were put through a series of washes (one 5-min wash at 37°C in 2xSSC, two 5-min washes at 37°C in 50% formamide/2xSSC, two 5-min washes at RT in 2xSSC). For chromosome painting, paint (Cytocell) was applied to the slide at 72°C for 2 min, then left overnight at 37°C in humidified chamber. The following day, slides were washed once with 0.4xSSC at 72°C for 2 min, then 2xSSC/0.05% Tween at RT for 30 s. After either staining method, slides were then stained with DAPI, then coverslips with Vectashield were applied and sealed.

### Microscopy

Images were acquired using an Olympus DeltaVision RT microscope (Applied Precision, LLC) equipped with a Coolsnap HQ camera. Three-dimensional image stacks were acquired in 0.2-μm steps, using Olympus ×100 (1.4 numerical aperture), ×60 or ×40 UPlanSApo oil immersion objectives. H2B-RFP-labelled cells were imaged in a four-well imaging dish (Greiner Bio-one). Twenty micrometer z-stacks (10 images) were acquired using an Olympus ×40 1.3 numerical aperture UPlanSApo oil immersion objective, every 3 min for 8 h using the DeltaVision microscope in a temperature- and CO_2_-controlled chamber. Deconvolution of image stacks and quantitative measurements was performed with SoftWorx Explorer (Applied Precision, LLC). Analysis was performed using Softworx Explorer.

### Single-cell sequencing

Samples from control and experimentally induced aneuploid cells were sorted by FACS prior to next-generation sequencing library preparation and data analysis using AneuFinder as previously reported [31, 61] except that the Strand Seq library preparation protocol [62] was used to create higher complexity libraries. Single nuclei were isolated and stained with 10 μg/mL propidium iodide and 10 μg/mL Hoechst. Single nuclei with low Hoechst/PI fluorescence (G1 population) were sorted into 96-well plates containing freezing buffer using a FACSJazz (BD Biosciences). Pre-amplification-free single-cell whole genome sequencing libraries were prepared using a Bravo Automated Liquid Handling Platform (Agilent Technologies, Santa Clara, CA, USA), followed by size-selection and extraction from a 2% E-gel EX (Invitrogen). Single-end 84-nt sequence reads were generated using the NextSeq 500 system (Illumina, San Diego, CA, USA) at 192 single-cell DNA libraries per flow cell. Demultiplexing based on library-specific barcodes and conversion to fastq format was done using bcl2fastq (v1.8.4, Illumina). Duplicate reads were called using BamUtil (v1.0.3). Demultiplexed reads were aligned to the GRCh38 reference genome using bowtie (v2.2.4), and only uniquely mapped reads (MAPQ>10) were used for further analysis. Copy number annotation was performed using AneuFinder (v1.4.0). Sequence reads are determined as non-overlapping bins with an average length of 500 or 40 kb, a GC correction is applied, and binned sequences are analyzed using Hidden Markov model, or Edivisive to determine the most likely copy number states. To negate the inherent sample variation introduced by sequencing single cells, a stringent quality control step was included that uses multivariate clustering to exclude libraries of insufficient quality. Chromosome copy number is plotted as a genome-wide state with clustering of cells based on the similarity of copy number profiles. To refine the breakpoints of 500-kb bin detected CNAs based on the position of 40-kb bin analysis, we used ClonalMasker to identify the same CNAs between 500 and 40 kb analyses and return the 40-kb breakpoints (see Fig. S3e for CNA pileups before and after refinement).

### Removal of clonal and sub-clonal CNAs

CNAs were considered to be clonal if they shared the same ploidy and their position in the genome overlapped by at least 50%. Using this criteria, a CNA was considered clonal if it appeared in more than two of the control (DMSO or siControl) cells. These CNAs were then filtered out from the experimentally induced CNAs for subsequent analysis. In addition, CNAs that occurred at the termini of chromosomes that were less than 1 Mb, or that occurred within, or in close (<1 Mb) proximity to centromeres were removed to prevent potential repetitive sequence-based mapping artifacts. CNAs in chromosome 12 in RPE1 cells were manually curated due to the frequent presence of trisomy 12. Lastly, cells with over 10 CNAs were removed from the analysis (except for siRNA experiments). The boundaries of low-resolution (500 kb) CNAs were refined using high-resolution (40 kb) CNAs by taking each low-resolution CNA and resetting its left breakpoint to be the left breakpoint of the leftmost overlapping high-resolution CNA, and similar for the right breakpoint. Both refinement and clonal CNA filtering scripts are available at https://github.com/MBoemo/clonalMasker. Final CNA lists are provided in Additional file 2 .

### Measurement of distance to large or giant genes, CFS or ERFS

For small CNAs—the distance from each breakpoint was calculated to the nearest end of the nearest large or giant gene. The closest distance was then plotted. For large terminal CNAs, only the interstitial breakpoint (and not the end of the chromosome) was used to calculate smallest distance to the closest large or giant gene. A list of large and giant genes (with their lengths and positions) was assembled from UCSC genome browser (GRCh38). Locations of CFS and ERFS were taken from the literature [33, 39, 42].

### Generation of randomly placed control regions

Random genomic coordinates and 1-Mb intervals were generated using the bedtools random script from the BEDTools software [63]. In total, 500 random coordinates were generated, 470 and 376 of which could be mapped to the genome during analyses of gene expression and replication timing respectively.

### RNA isolation, sequencing and analyses

Total RNA was extracted (RNeasy kit, Qiagen) from BJ and RPE1 cells, treated with either DMSO or aphidicolin, from three biological replicates. RNA quality was analyzed on Tapestations, with RIN numbers above 7.0. Library preparation was performed using the Lexogen 3′ tagSeq kit. RNA sequencing was performed by Barts and the London Genome Centre on the Illumina NextSeq 500 platform, generating on average ~10 million single-end reads of 76 bp in length per sample. Raw reads were mapped to the human genome (hg38, Genome Reference Consortium GRCh38) using HISAT2 [64]. Number of uniquely aligned reads (q > 10) to the exonic region of each gene were counted using HTSeq [65] based on the GenCode annotation release 29. Only genes that achieved at least one read count per million reads (cpm) in at least three samples were kept and a log2 transformed cpm expression matrix was subsequently generated. Differential expression analysis was performed using the “limma” R package [66]. In order to analyze total gene expression levels at aCNAs we generated a 2-Mb window of analysis centered around the putative breakpoint. The average expression of all genes in that window was summed. These values were then plotted in categories of small (<20 Mb) or large (>20 Mb) gain and loss CNAs. For large and giant gene expression analysis, we used the mean gene expression from three biological replicates.

### Replication timing

For replication timing, both single and bulk G1 cells serving as controls were sorted. For experimental samples, we sorted various S-phase populations based on DNA content (late G1/early S; early S/mid S; mid S/late S; late S/early G2). Single-cell sequencing was performed, and reads were processed as described above. The replication timing method as described by Takahashi [42] was then applied. Uniquely mappable sequence reads were binned into 1-Mb bins. Read counts per bin were then determined and converted into a read proportion across all reads. Bins with a read proportion lower than the 0.1 quantile were excluded. Quantiles were calculated separately for the autosomes and X-chromosome in male samples (the Y-chromosome was not included, neither were aneuploid chromosomes). To correct for variable mappability, median centering was applied using the data from G1 cells as a reference. Correction factors were calculated by dividing the average read proportion by the proportion of each bin. Bin proportions were then multiplied by the correction factors to yield a corrected average proportion per bin. To determine whether a bin was replicated or not, a quantile cut-off was applied per S-phase population to reflect the fraction of the genome expected to be replicated at that moment (i.e., 0.125, 0.375, 0.635, and 0.875 for the population mentioned above). Bins with read proportion greater than the quantile cut-off set per S-phase population were designated as “replicated” (blue in the plots). Conversely, bins with proportions below the quantile cut-off were designated “unreplicated” (yellow in the plots).

### Replication timing factor

For each bin, we determined whether the genome was replicated or not (1= replicated, 0= not replicated), in each of the single cells, at each of the four replication phases (early S-phase, mid S-phase, mid-late S-phase, and late S-phase). We then summed the values across all replication phases, creating the replication factor value, where higher values represent earlier replication. We then categorized the range of replication factor values in four quartiles. Lowest quartile represents late replicated bins and highest quartile indicates bins that were replicated in early S-phase, with mid-early and mid-late quartiles in between the two.

### SNP 6.0 analysis

SNP 6.0 analysis was performed by Aros AB (Denmark) using a Genome-Wide Human SNP Array 6.0 (Affymetrix) on RPE1 cells and two edited RPE1 lines derived from the parental RPE1 line. Data were analyzed in the Chromosome Analysis Suite (CAS, Affymetrix). Data were transformed from global references obtained from signals in the CAS normalized reference library.

### Breakpoint analysis

Large and small CNAs were separated into breakpoints with an R [67] script. The BED files containing the features of interest and breakpoints were mapped to the RT bins with the BEDTools software [63]. Bins at the beginning and end of each chromosomes were removed. Breakpoints in bins with and without features of interest were counted in R with Tidyverse packages [68] and compared to the distribution of bins with a Chi-Square Goodness of Fit Test, dropping any comparison with small (*n* <5) groups.

### Secondary structure repeat analysis

To predict regions of the genome prone to breakage due to secondary structure formation, motif cluster analysis was conducted for repetitive sequences previously predicted to be structure-forming and associated with breakpoints under ATR inhibition and aphidicolin treatment [48]. Genomic regions enriched for these sequences were identified by searching each reported sequence as a motif against the hg38 reference genome using MCAST software [69] with significance threshold of e-value<100. Any overlapping results were combined to form a single region. We then calculated the % of each RT bin covered by the repeats.

### Statistical analysis

Unpaired *t*-test or one-way ANOVA Kruskal-Wallis test with post hoc Dunn’s correction were used to test for levels of significance using either Excel or Prism (GraphPad). Asterisks have been used to denote the significance value between experimental conditions adhering to the following nomenclature: *p*<0.05 (*); *p*<0.005 (**); *p*<0.0005 (***); *p*<0.00005 (****).

## Supplementary Information

Additional file 1. Contains supplementary Figs S1-S8.

Additional file 2. Contains CNAs for RPE1 after treatment with DMSO, Aphidicolin or siRNA.

Additional file 3. Replication Timing factor derived from RPE1 single cells after treatment with DMSO or aphidicolin.

Additional file 4. RNAseq data for BJ and RPE1 cells treated with DMSO or aphidicolin.

Additional file 5. Review history.

## Data Availability

Raw single-cell sequencing reads are available at European Nucleotide Archive using accession number PRJEB56042 [70]. RNA-seq data is available at NCBI Gene Expression Omnibus, using accession number GSE168689 [71]. Raw microscopy images have been uploaded to Figshare [72].
