# Supplementary material for: Replication stress generates distinctive landscapes of DNA copy number alterations and chromosome scale losses
Source: Genome Biol. 2022 Oct 20;23:223. doi: 10.1186/s13059-022-02781-0 (PMC9583511; doi:10.1186/s13059-022-02781-0)
Supplement: Supplementary file 1 — Additional file 1. Contains supplementary Figs S1-S8. [file 13059_2022_2781_MOESM1_ESM.docx]

**Replication stress generates distinctive landscapes of DNA copy number alterations and chromosome scale losses**

**Nadeem Shaikh**†**, Alice Mazzagatti**†**, Simone De Angelis, Sarah C. Johnson, Bjorn Bakker, Diana C.J. Spierings, René Wardenaar, Eleni Maniati, Jun Wang, Michael A. Boemo, Floris Foijer, Sarah Elizabeth McClelland**

Supplemental Figures and Figure legends.

**
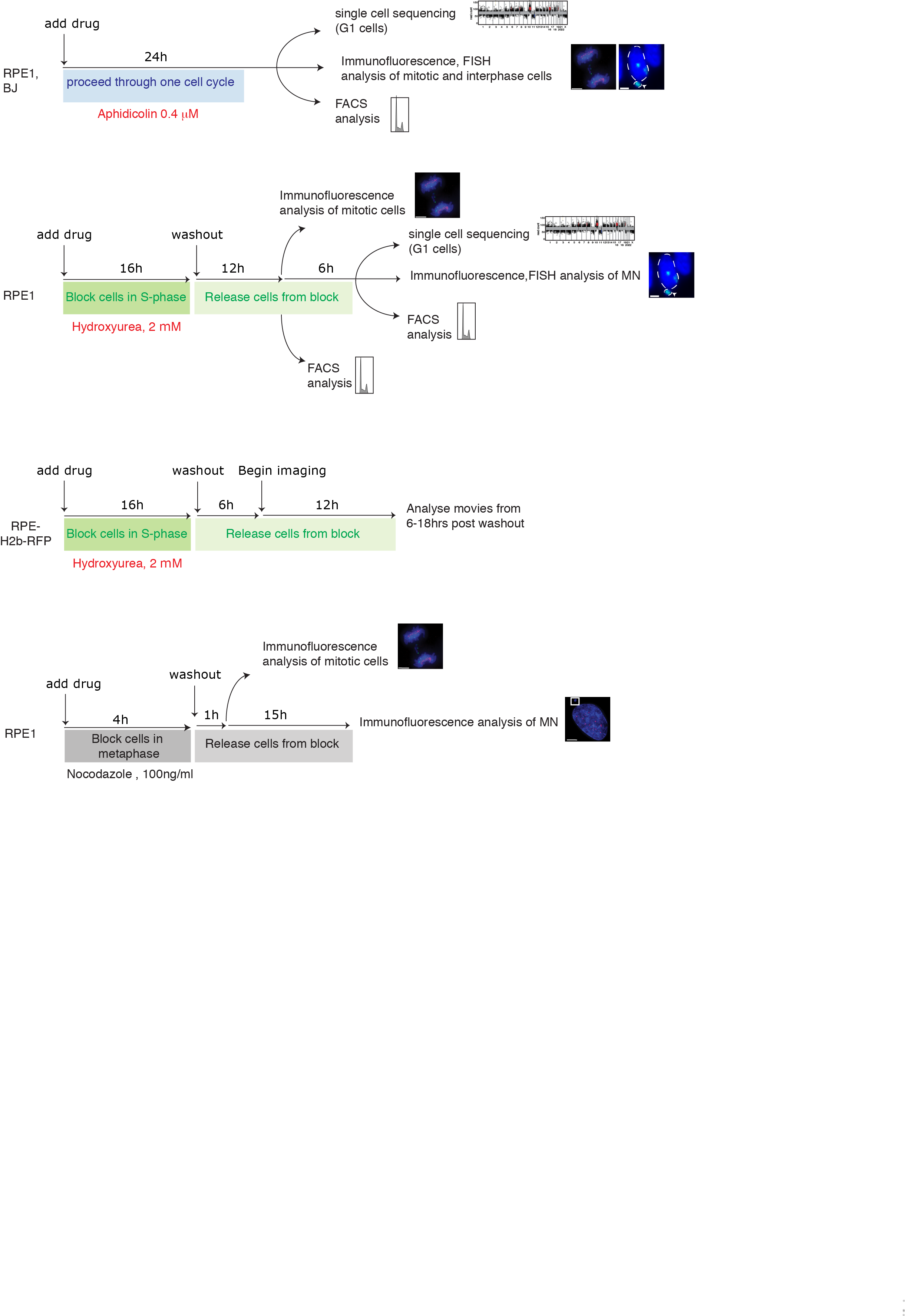
**

**Fig S1:** Schematic of workflow for experiments to collect treated cells for sequencing, FACS analysis or staining by immunofluorescence or FISH.

**
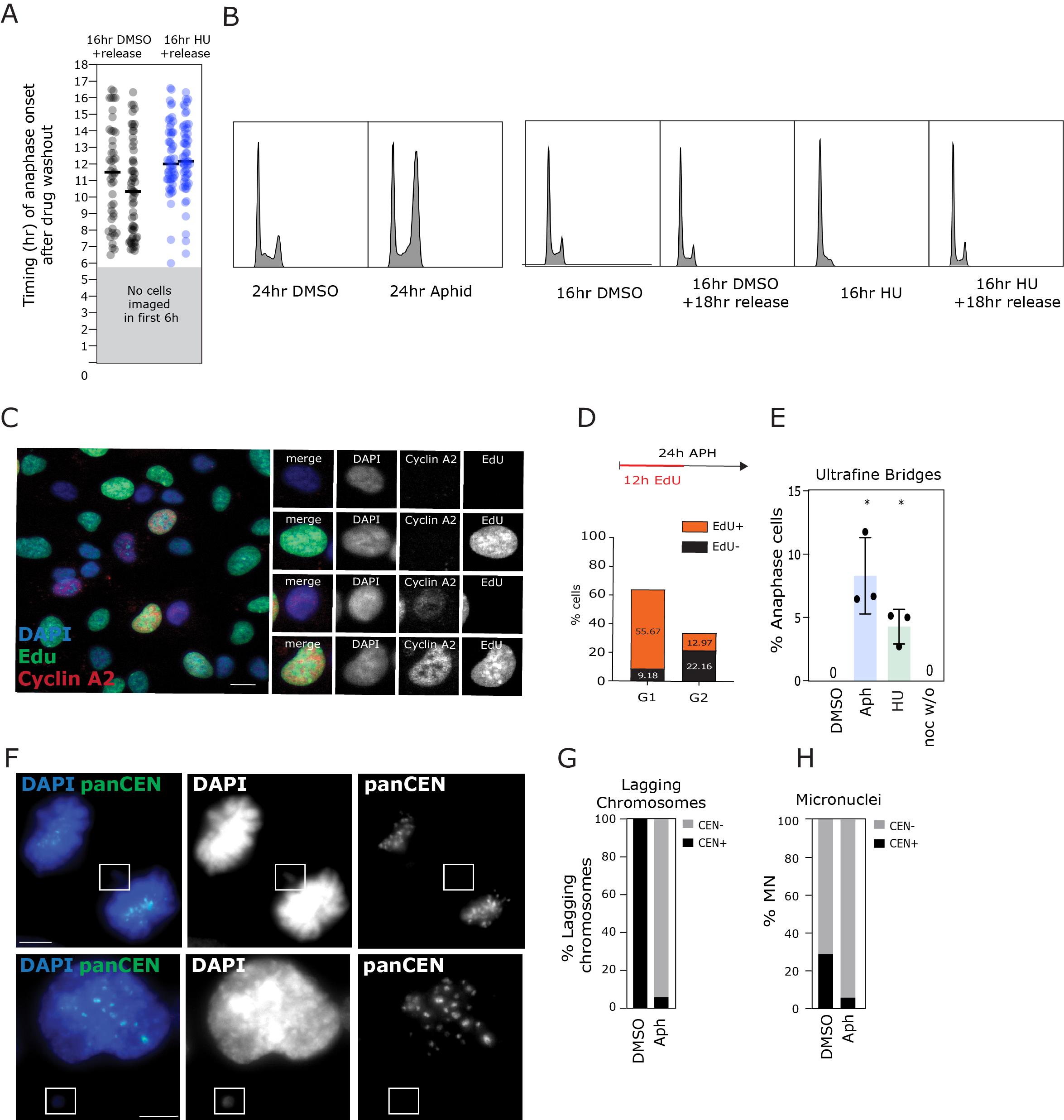
**

**Fig S2: (relating to Figure 1)** (**A,B**) Live cell imaging (A) of RPE1 cells expressing fluorescently tagged histones (RPE1-hTERT H2B-RFP) reveals enrichment of cells entering mitosis at 10-12hr timepoint in HU treatment, compared to DMSO cells entering mitosis at steady state; and Fluorescence activated cell sorting (B) revealed that HU block resulted in a depletion of the G2 population which recovered after release. Both are consistent with an efficient enrichment of cells in early S-phase. (A) shows summary of two experiments, (B) images are representative of two experiments. (**C**) Images of RPE1 cells stained for Cyclin A and EdU incorporation. RPE1 cells were treated with DMSO or aphidicolin for 24 h, including a 12 h pulse of EdU as indicated. (**D**) EdU incorporation into Cyclin A positive (S/G2) or negative cells (G1) was then quantitated from immunofluorescence microscopy images (n=185 and 235 cells). **(E)** Quantification of RPA-coated UFBs in RPE1 cells, summary of three experiments, at least 30 anaphases per condition per experiment. **(F)** Representative images of RPE1 anaphase cell with a segregation error or an interphase cell with micronucleus, after 24hr treatment with aphidicolin, and probed with a pan-centromeric FISH probe. (**G**),(**H**) Centromere status of lagging chromosomes or micronuclei after indicated treatments, based on pan-centromeric FISH probe staining (16-29 lagging chromosomes, 104-109 MN scored).

**
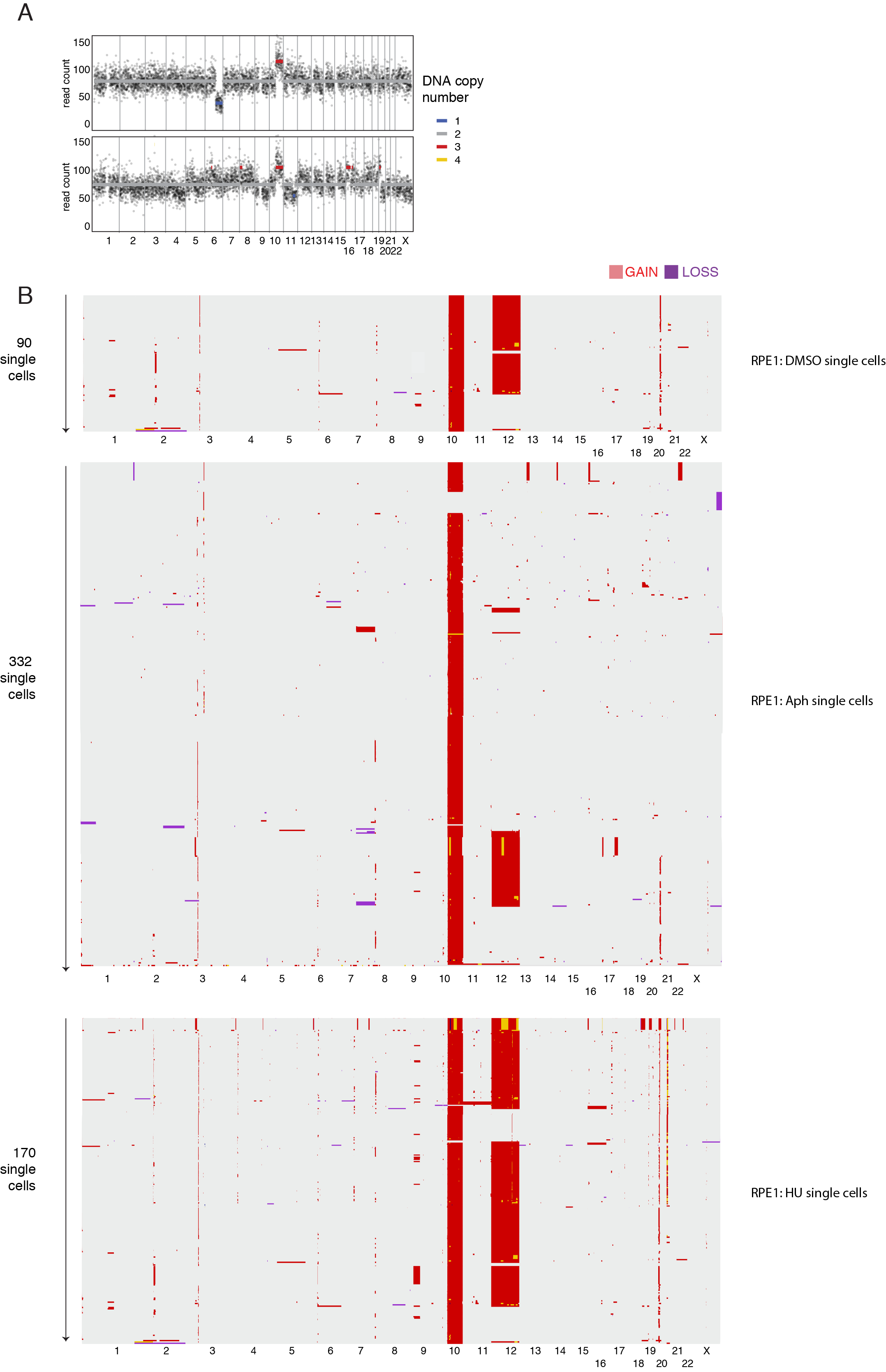
**

**Fig S3 (related to Figure 2):** (**A**) Example of Single cell sequencing readout. Coloured bars represents average copy number for those reads. (**B**) Upper panel: single cell sequencing data from 90 RPE1 cells treated with DMSO; purple represents copy number losses, red represents gains. Amplification of 10q and recurrent aneuploidy of chromosome 12 are known aberrations in the RPE1 line. Middle panel; single cell sequencing of 332 RPE1 cells treated with aphidicolin. Lower panel; single cell sequencing of 170 cells treated with hydroxyurea. Recurrent focal amplifications were observed in both control, aphidicolin and hydroxyurea-treated cells. We reasoned that these likely represented existing structural variations present at clonal or sub-clonal frequencies and removed these from our analyses (methods).

**
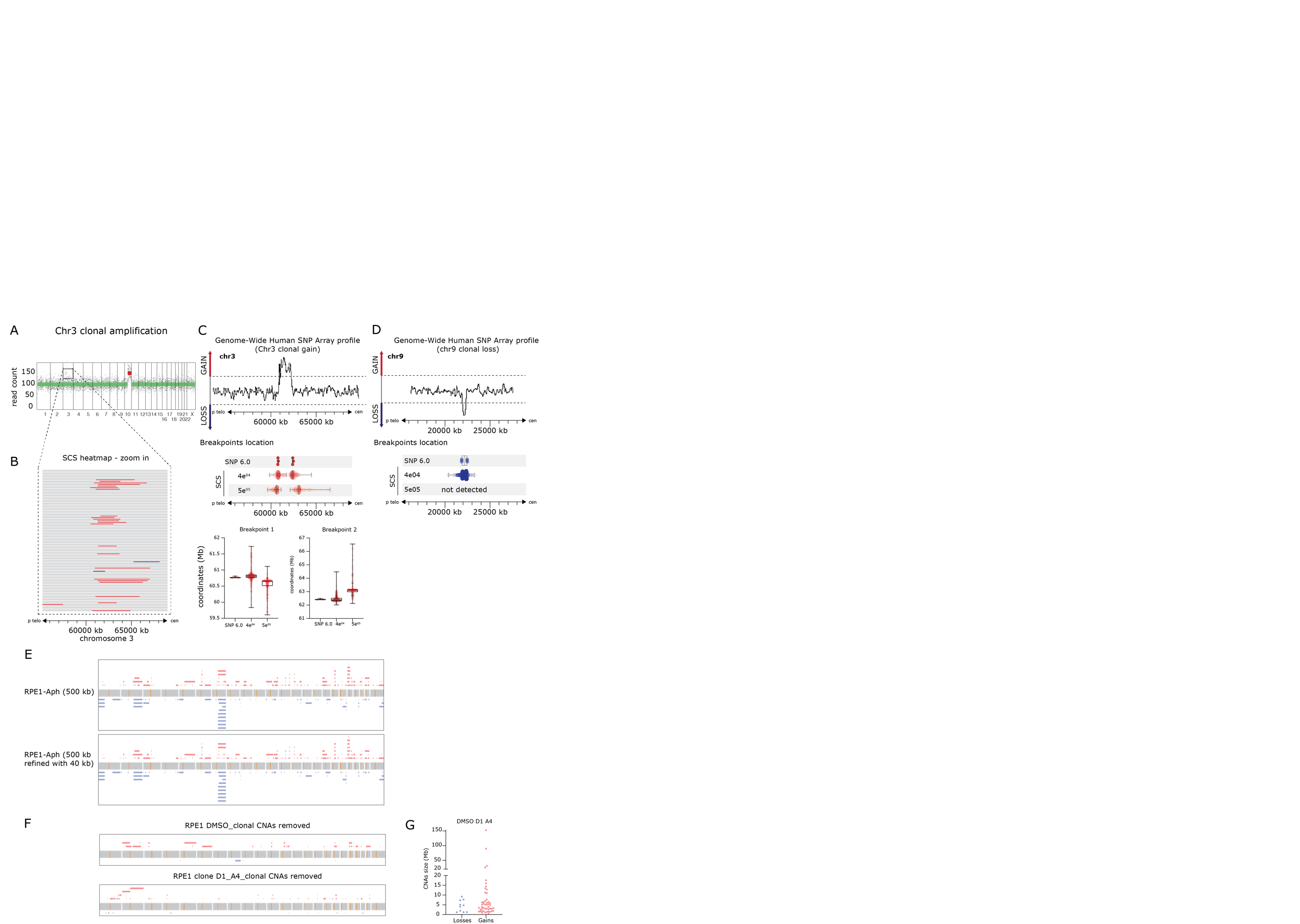
**

**Fig S4 (related to Figure 2):** (**A,B**) Schematic indicating SNP 6.0 array verification of clonal focal amplification on chromosome 3 in RPE1 cells, and comparison between 500 kb and 40 kb binning CNA detection accuracy. The three SNP 6.0 datapoints indicate CNA positions determined from three independent RPE1 cell line clones analysed by SNP 6.0 array in parallel. To benchmark the resolution of CNA detection using single cell sequencing, we analysed copy number alterations present in the parental RPE1 cell line using a SNP 6.0 array which verified the presence of a ~1.6 Mb focal amplification at chromosome 3p14.2 present in the majority of RPE1 cells. (**C,D**) A higher resolution copy number analysis using 40 kb binning (see **Methods**) was able to detect smaller bona fide CNAs (~500 kb) that were also visible from SNP 6.0 array data, with higher accuracy of breakpoint position However, a large number of likely artefactual CNAs were also observed with this analysis in both DMSO and aphidicolin-treated cells. Therefore, we maintained the original (500 kb binning) resolution analysis to identify bona fide CNAs, but refined the position of these using the 40 kb binned analysis in order to pinpoint CNA breakpoints with the highest accuracy (**Methods**). (**D**) Detection of ~ 0.5 Mb clonal loss with 40 kb but not 500 Kb binning CNA detection. (**E,F**) Pileups of all detected aCNAs (after removing clonal events, centromeric and telomeric events (see **Methods**)) before (top panels) and after (lower panels) refining breakpoint position using 40 kb calls (see **Methods**) for RPE cells. (**G**) Pileups of all detected CNAs (after removing clonal events as in E,F) in RPE1 population (top panel) or a population derived from a single cell (lower panel) treated with DMSO. (**H**) CNA sizes from the D1_A4 RPE1 clone.

**
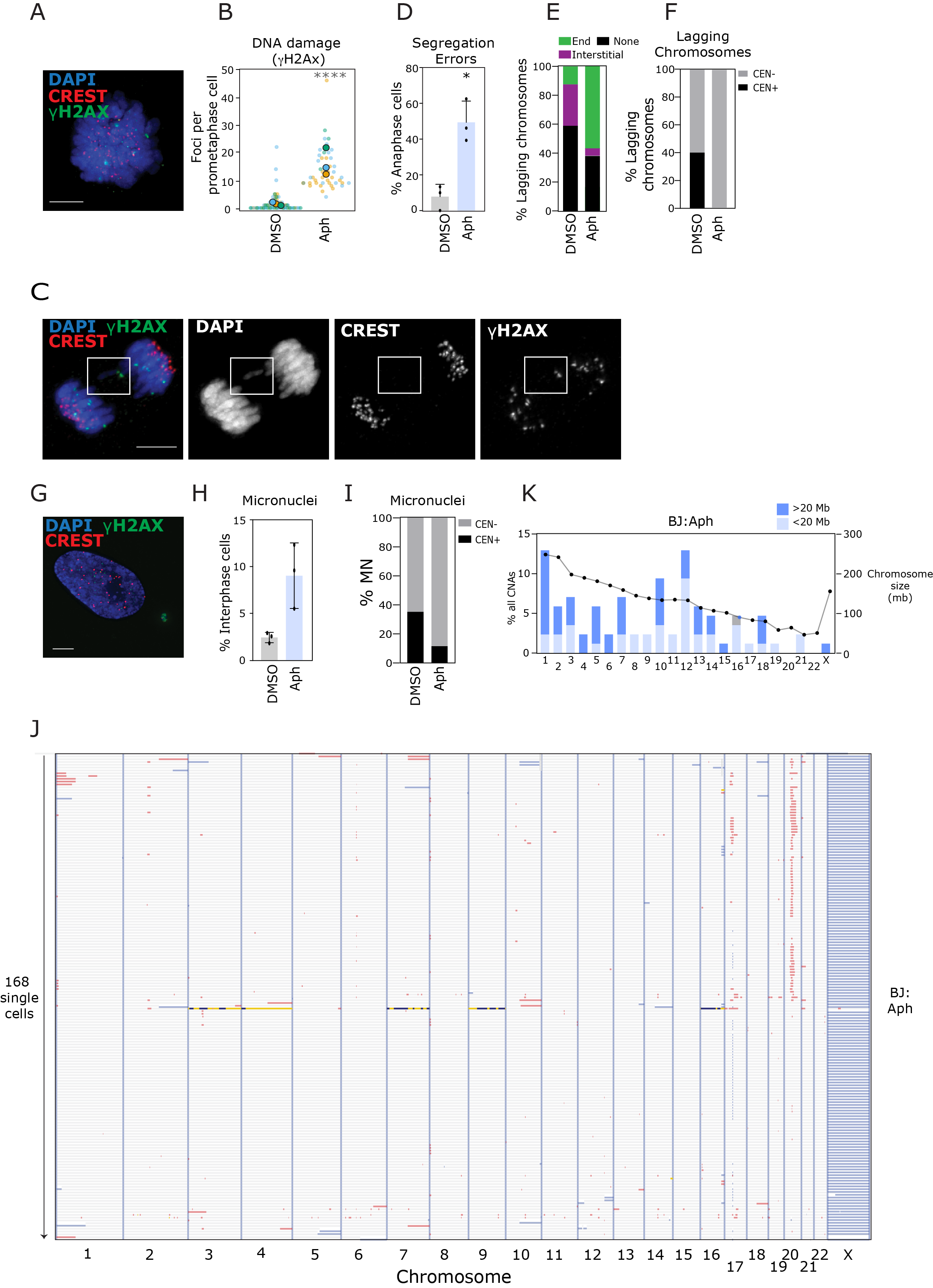
**

**Fig S5: (relating to Figure 3**): **(A)** Immunofluorescence images of BJ prometaphase cells. **(B)** Quantification of γH2Ax DNA damage foci in BJ cells after indicated treatments (n=66 and 73 cells). Each individual experiment and its matching mean value is in a separate colour. **(C)** Representative images of BJ anaphase cells with segregation errors, with DNA damage and centromere status indicated by γH2AX and CREST staining . **(D)** Quantification of segregation errors in BJ anaphase cells after 24h treatment with DMSO or 0.4 μM aphidicolin. **(E)** Quantification of DNA damage on lagging chromosomes in BJ cells. **(F)** Centromere status of lagging chromosomes in BJ cells, based on CREST staining. **(G)** Image of BJ interphase cell with micronucleus **(H)** MN rates in BJ cells after indicated treatments (n=429 and 482 cells from three experiments). **(I)** Centromere status of BJ micronuclei. (**J**) Single cell sequencing results of BJ cells treated with 0.4 μM aphidicolin for 24h. (**K**) Frequencies of CNAs (left y-axis) across each chromosome after aphidicolin treatment in BJ cells, divided into large (>20 Mb) and small (< 20 Mb) CNAs. Dotted line indicates relative size of chromosomes (right y-axis).

Statistical tests are unpaired *t*-tests.


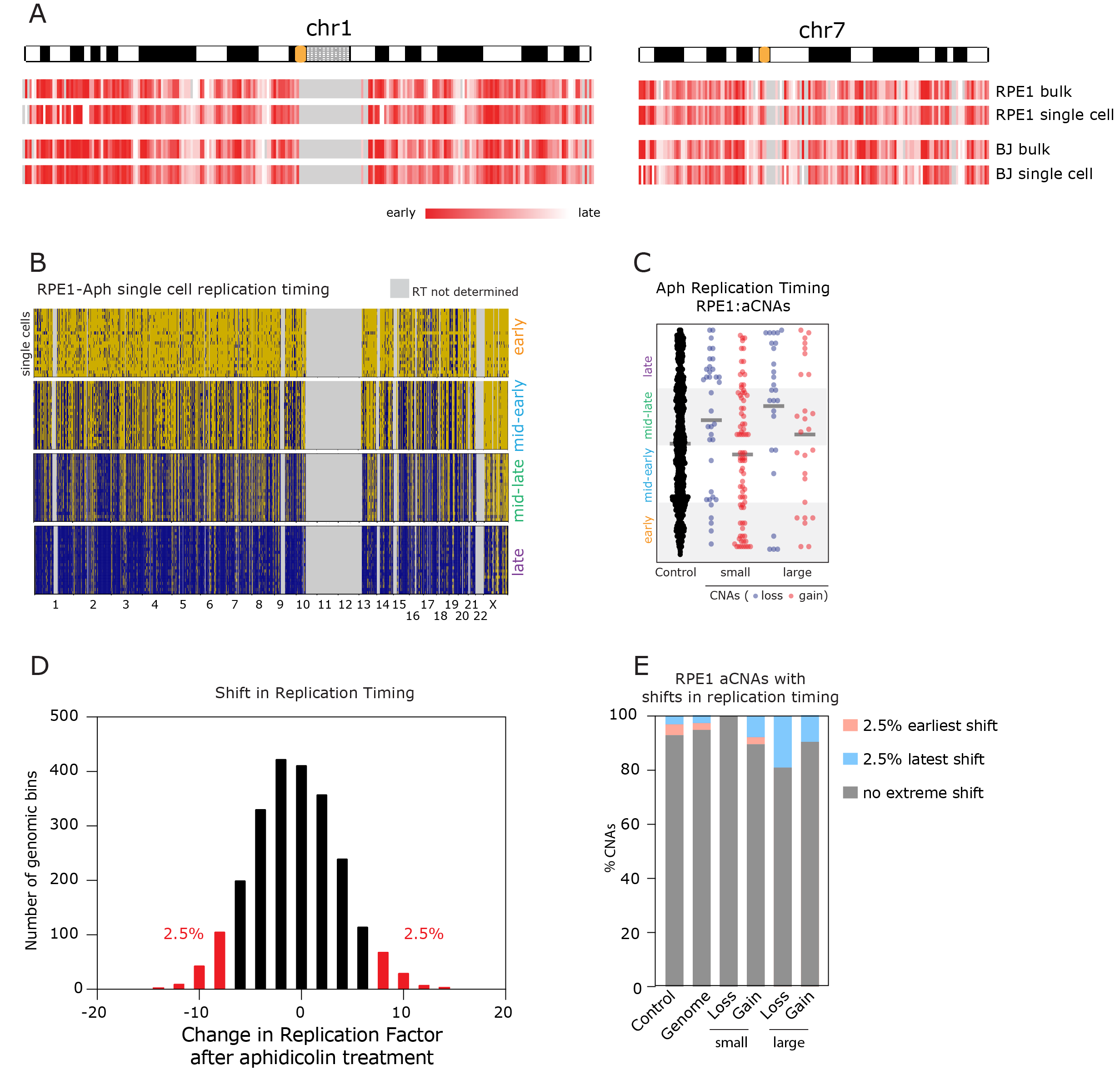


**Fig S6: (relating to Figure 3**): (**A**) Comparison between single cell and bulk replication timing for RPE1 and BJ cells for two selected chromosomes. (**B**) Single cell replication timing analyses for RPE1 cells during aphidicolin treatment, from each S phase fraction as indicated. Dark blue indicates replicated genomic regions. (**C**) Replication Timing factor for random control or aCNAs in RPE1 measured in cells treated with aphidicolin. (**D**) Analysis of genomic regions in RPE1 that shift from earlier or later in their replication timing, after aphidicolin treatment. Red bars indicating the genomic bins with greatest shift of early to later or late to earlier replication timing (the upper 2.5% and lower 2.5% of all genomic bins). (**E**) Proportion of aCNAs in RPE1 that were positioned in 1 Mb genomic bins with the greatest shift in replication timing. This is in comparison to random in silico control regions or how the genomic bins themselves changed.

**
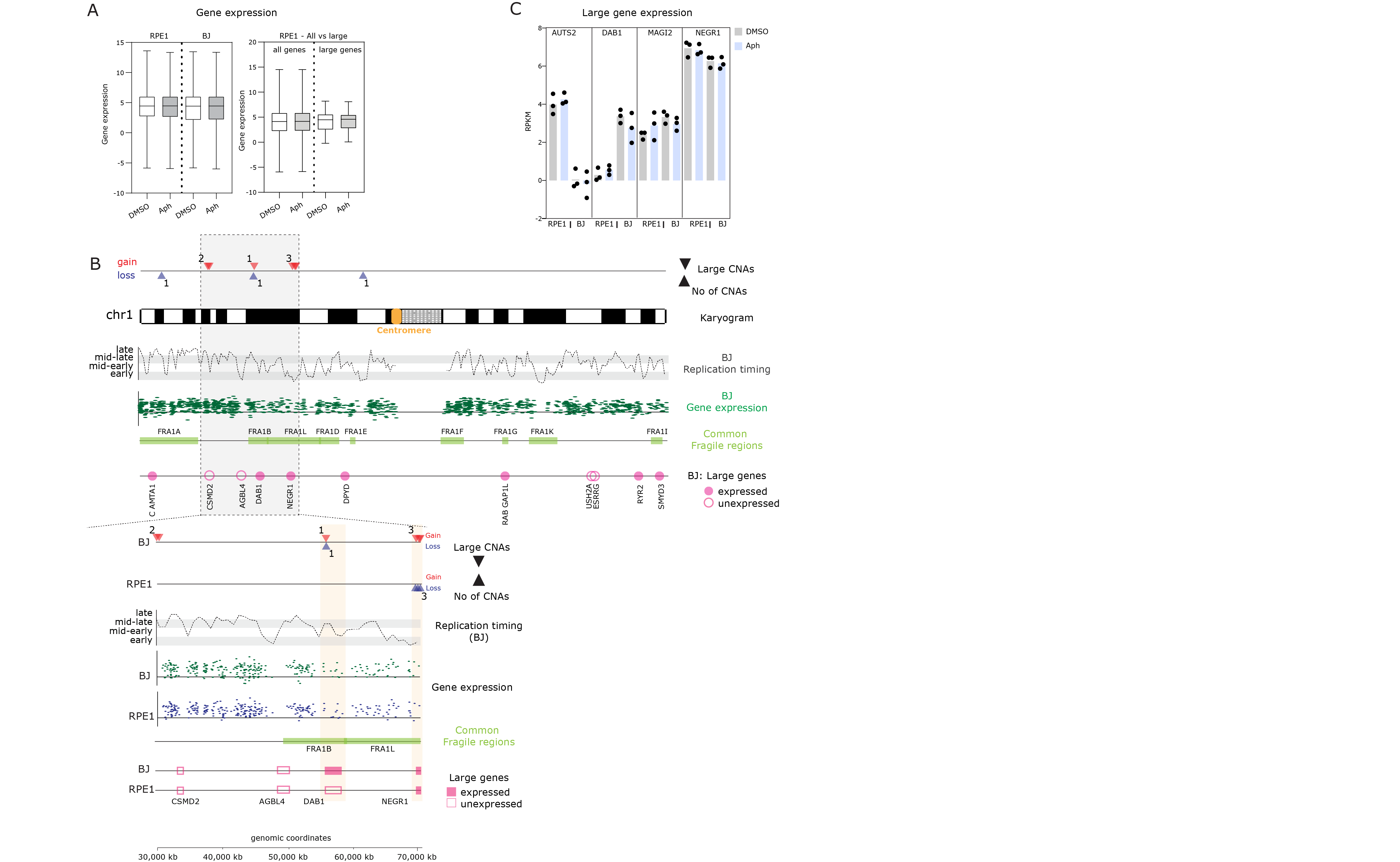
**

**Fig S7 (related to Figure 4): (A)** Left panel: Total gene expression in DMSO vs aphidicolin for RPE1 and BJ cells; right panel: expression in RPE1 cells treated with DMSO vs aphidicolin for all genes vs only large genes (>600 kb). **(B)** Schematic of chromosome 1, with position of CNAs found in BJ cells, with replication timing profile, gene expression (each dot indicates location and expression level of an individual gene), location of human chromosome 1 CFSs compiled from literature, location and expression status of large genes. Zoom of selected portion indicates genomic characteristics and CNA positions in BJ and RPE1 cells. (**C**) Expression levels of four large genes in RPE1 and BJ cells in both DMSO and aphidicolin treatments (summary of three experiments). Within each cell line, there was no significant difference in expression for each gene in DMSO or aphidicolin (t-test).

**
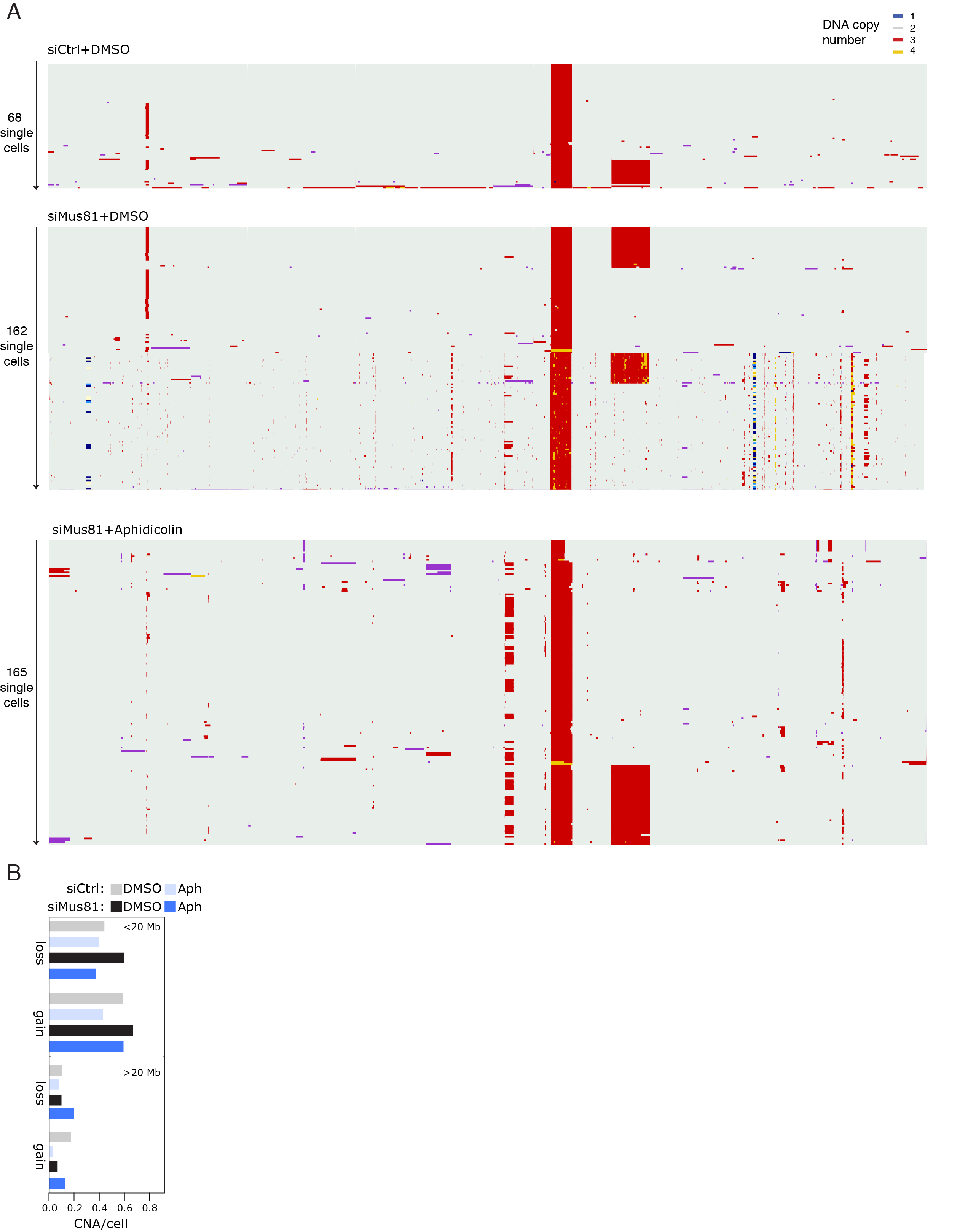
**

**Fig S8 (related to Figure 6): (A)** Heatmaps indicating CNAs detected by Single cell sequencing of RPE1 cells treated with siCtrl or siMus81 in DMSO or aphidicolin as indicated. (**B**) CNA rates per cell in RPE1 cells under indicated conditions.
