# Supplementary material for: Replication stress generates distinctive landscapes of DNA copy number alterations and chromosome scale losses
Source: Genome Biol. 2022 Oct 20;23:223. doi: 10.1186/s13059-022-02781-0 (PMC9583511; doi:10.1186/s13059-022-02781-0)
Supplement: Supplementary file 5 — Additional file 5. Review history. [file 13059_2022_2781_MOESM5_ESM.docx]

Review History

**First round of review**

**Reviewer 1**

**Were you able to assess all statistics in the manuscript, including the appropriateness of statistical tests used?** Yes, and I have assessed the statistics in my report.

**Comments to author:**

In this manuscript, authors used single cell sequencing to determine DNA copy number alterations (CNAs) that are induced by replication stress after one cell cycle. The study is performed in the absence of any selective pressure, which provides new information beyond previous study to detect CNAs that survive clonal outgrowth. However, some findings are surprising including very few recurrent CNAs at CFSs after aphidicolin treatment. This work would be potentially of interest to the field if major concerns are addressed.

1. It has been shown by different approaches that CFSs are the hot spots for chromosomal rearrangements and CNAs upon aphidicolin treatment. Hydroxyurea (HU) treatment causes breakage at early replicating fragile sites (ERFSs) that are distinctive from CFSs1. It will be informative to align all CNAs identified in this study with CFSs and ERFSs. Authors claimed that "Different replicative stresses generated distinct profiles of CNAs". However, only aphidicolin treatment is shown in this study.

2. It has been shown that DNA secondary structures are often hot spots for chromosomal breakage upon replication stress2. In this study, is there an overlap of mapped CNAs with DNA secondary structures?

3. It is hard to find the concentration of aphidicolin used in this study for BJ and RPE cells. The concentration should be clearly indicated in the figure legends. It is important to show that single cell sequencing was carried out using the same concentration of aphidicolin and under the same condition that would induce CFS expression in BJ and RPE cells.

4. It needs to show DNA replication timing and transcription profiles after aphidicolin treatment. This will provide additional information to understand the recurrent sites for CNAs in BJ and RPE cells.

5. MUS81 was shown to actively cleave underreplicated regions that are often present at CFSs3,4 to prevent chromosome bridging. It is surprising to see the features of CNAs in MUS81 knockdown cells are different from that in aphidicolin treated cells. To confirm the results, MUS81 knockout cells should be used. It is also important to show CNAs in MUS81 knockout cells after aphidicolin treatment, which would address whether the observed CNAs induced by aphidicolin are dependent on MUS81.

References:

1. Barlow, J.H. et al. Identification of early replicating fragile sites that contribute to genome instability. Cell 152, 620-32 (2013).

2. Shastri, N. et al. Genome-wide Identification of Structure-Forming Repeats as Principal Sites of Fork Collapse upon ATR Inhibition. Mol Cell 72, 222-238 e11 (2018).

3. Naim, V., Wilhelm, T., Debatisse, M. & Rosselli, F. ERCC1 and MUS81-EME1 promote sister chromatid separation by processing late replication intermediates at common fragile sites during mitosis. Nat Cell Biol 15, 1008-15 (2013).

4. Ying, S. et al. MUS81 promotes common fragile site expression. Nat Cell Biol 15, 1001-7 (2013).

**Reviewer 2**

**Were you able to assess all statistics in the manuscript, including the appropriateness of statistical tests used?** No, I do not feel adequately qualified to assess the statistics.

**Comments to author:**

In the manuscript "DNA Replication Stress Generates Distinctive Landscapes of DNA Copy Number Alterations and Chromosome Scale Losses"" Mazzagatti et al present data obtained from single cell sequencing that maps copy number alterations (CNAs) after one round of replication stress. They have used the non-transformed human cell lines RPE1-hTERT and BJ-hTERT. The authors analyse the CNAs according to replication timing and location of large and giant genes and complement their experiments with immunofluorescence assays.

Although single cell seq is an impressive technique this study does not significantly advances the understanding of replication-stress induced copy number alterations. Moreover, several claims are not adequately backed up by the data and finally citations and introduction to background literature are inaccurate.

Major points

1. Conclusions and interpretations are not adequately backed up

The conclusion that "large terminal CNAa but not small losses are associated with late replication timing" is surprising. It is clear from several published cancer genomic studies that small losses are associated with CFSs which in general have late replication timing. The authors should discuss why the method used here does not identify this well-established pattern.

In the figure text for fig 2 c, d and e it is important to write the total number of CNAs in each category. Is insufficient statistical power the reason that so few significant effects are observed throughout the paper?

Fig 1L. It is surprising that APH has such small effect in particular in RPE1 cells where most categories are underrepresented after APH treatment compared to DMSO. It is highly problematic for any conclusions that relate to replication stress-induced CNAs.

Fig. 3B.

What does the dots in gene expression actually represent? There is only on dot in AUTS2 -RPE cells. Does this mean that only one read was obtained?

When making any conclusions regarding expression of large and giant genes and their consequences for fragility it is very important to confirm that the entire gene is transcribed because the extreme size of the transcription unit is presumably the trigger for fragility. The authors should clearly indicate if they can confirm transcription throughout the large and giant genes, when they conclude whether or not transcription of large genes is a trigger for fragility. Also, it should be mentioned in the text if the depth used for RNAseq is sensitive enough to detect very low expression.

For Fig. 4 I the n numbers are very small. Was the experiment only carried out once?

It should be carried out three times and then the authors must quantify a decent number from each experiment.

In E it is not written what the error bars show.

Fig. 5. The authors should explain why they do not see any signs of replication stress-induced aberration in mitosis after Mus81 depletion when looking at gH2AX, anaphase errors or UFBs. Yet the single cell seq suggest that there are mitotic aberrations with MUS81 knockdown. - Could these arise in S phase rather than by errors in mitosis?

In the discussion the authors state that "…. In RPE cells Mus81 is not essential for convertion of fragile sites into chromosome breaks …". The statement is based on the observation that Mus81 depletion did not reduce acentrentic lagging chromosomes and micronuclei. My feeling is that the authors have misunderstood the message in the cited paper (51), because the point of this paper is that microscopically visible "chromosome breaks", are in fact not DNA breaks but represent a repair intermediate. Consistently, in this paper it was already shown that MUS81 depletion leads to increased micronuclei formation. (In addition, the authors should also cite the back-to-back paper from Naim et al NCB 2013).

2. The referencing is inadequate

Reference 1 and 15 is the same paper

In line 36-37, when mentioning oncogenes as drivers for replication stress why have the authors not cited the two hallmark papers here (Bartkova et al., 2005; Di Micco et al., 2006)?

In line 42-44 MiDAS is introduced but the authors do not cite the first papers reporting DNA synthesis in mitosis (MiDAS) (Minocherhomji et al., 2015; Pedersen et al., 2015). It is correct that the term was coined in the (Bhowmick et al., 2016) paper, which is thus correctly cited.

In line 45 the authors write "…. Replication stress can induce lagging chromatin, anaphase bridges, aneuploidy and micronuclei formation". Again, the reference for this statement is insufficient. There are several papers reporting on these phenomena that came out before the cited paper - for instance (Chan et al., 2009; Ichijima et al., 2010; Naim and Rosselli, 2009)

In line 53 the authors should also cite (Pentzold et al., 2018) for use of FANCD2 ChIPseq to map CFSs.

In the first result section 53BP1 NBs are analysed (Fig S1A). However, 53BP1 NBs have not been introduced and the authors have not cited the papers that first described 53BP1 here (Harrigan et al., 2011; Lukas et al., 2011).

Minor points.

The second sentence in the abstract does not make sense

Quantification of gH2AX foci in fig 1D and of 53BP1 in Fig S1 B and G and similar quantifications should be represented by a super plot as explained in https://rupress.org/jcb/article/219/6/e202001064/151717/SuperPlots-Communicating-reproducibility-and

It is not clear from figure or figure text what noc w/o refer to. It should be noted in the fig text or changed.

Line 41 p. Low levels of replication stress can bypass ATR (wrong to mention ATM here) as shown by Koundrioukoff et al.

Bartkova, J., Horejsi, Z., Koed, K., Kramer, A., Tort, F., Zieger, K., Guldberg, P., Sehested, M., Nesland, J.M., Lukas, C., et al. (2005). DNA damage response as a candidate anti-cancer barrier in early human tumorigenesis. Nature 434, 864-870.

Bhowmick, R., Minocherhomji, S., and Hickson, I.D. (2016). RAD52 Facilitates Mitotic DNA Synthesis Following Replication Stress. Mol Cell 64, 1117-1126.

Chan, K.L., Palmai-Pallag, T., Ying, S., and Hickson, I.D. (2009). Replication stress induces sister-chromatid bridging at fragile site loci in mitosis. Nat Cell Biol 11, 753-760.

Di Micco, R., Fumagalli, M., Cicalese, A., Piccinin, S., Gasparini, P., Luise, C., Schurra, C., Garre, M., Nuciforo, P.G., Bensimon, A., et al. (2006). Oncogene-induced senescence is a DNA damage response triggered by DNA hyper-replication. Nature 444, 638-642.

Harrigan, J.A., Belotserkovskaya, R., Coates, J., Dimitrova, D.S., Polo, S.E., Bradshaw, C.R., Fraser, P., and Jackson, S.P. (2011). Replication stress induces 53BP1-containing OPT domains in G1 cells. J Cell Biol 193, 97-108.

Ichijima, Y., Yoshioka, K., Yoshioka, Y., Shinohe, K., Fujimori, H., Unno, J., Takagi, M., Goto, H., Inagaki, M., Mizutani, S., et al. (2010). DNA lesions induced by replication stress trigger mitotic aberration and tetraploidy development. PLoS One 5, e8821.

Lukas, C., Savic, V., Bekker-Jensen, S., Doil, C., Neumann, B., Pedersen, R.S., Grofte, M., Chan, K.L., Hickson, I.D., Bartek, J., et al. (2011). 53BP1 nuclear bodies form around DNA lesions generated by mitotic transmission of chromosomes under replication stress. Nat Cell Biol 13, 243-253.

Minocherhomji, S., Ying, S., Bjerregaard, V.A., Bursomanno, S., Aleliunaite, A., Wu, W., Mankouri, H.W., Shen, H., Liu, Y., and Hickson, I.D. (2015). Replication stress activates DNA repair synthesis in mitosis. Nature 528, 286-290.

Naim, V., and Rosselli, F. (2009). The FANC pathway and BLM collaborate during mitosis to prevent micro-nucleation and chromosome abnormalities. Nat Cell Biol 11, 761-768.

Pedersen, R.T., Kruse, T., Nilsson, J., Oestergaard, V.H., and Lisby, M. (2015). TopBP1 is required at mitosis to reduce transmission of DNA damage to G1 daughter cells. J Cell Biol 210, 565-582.

Pentzold, C., Shah, S.A., Hansen, N.R., Le Tallec, B., Seguin-Orlando, A., Debatisse, M., Lisby, M., and Oestergaard, V.H. (2018). FANCD2 binding identifies conserved fragile sites at large transcribed genes in avian cells. Nucleic Acids Res 46, 1280-1294.

**Reviewer 3**

**Were you able to assess all statistics in the manuscript, including the appropriateness of statistical tests used?** No, I do not feel adequately qualified to assess the statistics.

**Comments to author:**

The manuscript Mazzagatti et al describes using a fairly novel technology of single cell sequencing to detect DNA copy number alterations (CNAs) to show how replication stress causes focal CNAs and chromosomal alterations (small and large scale gains or losses). The authors use two different diploid non-transformed cell lines for their analysis and carefully controlled for ensuring that the CNA analysis was caused by one cell cycle after undergoing replication stress. Although the results of the study was not unexpected (recurrent CNAs were largely confined to large genes that are somewhat transcribing), the potential of using this type of platform to study cancer genome evolution trajectories is intriguing. My only concern is the heavy reliance on low dose Aphidicolin (APH) for generating their single-cell CNA analysis. As it is well-understood that APH works to predominantly and irreversibly block Polalpha (lagging strand synthesis) activity, and also leading-strand, processive DNA polymerases (Pol delta/epsilon), this completely disrupts replication forks, and therefore requires the use of backup or dormant origins to fulfill DNA synthesis during S-phase in a timely manner. So low dose APH treatment is essentially a stochastic method of randomly blocking forks throughout the genome. So it is very obvious why these would be disproportionately affecting large genes in the human genome, as most replication forks (origin initiation) occurs at or near Transcription start sites of actively transcribing regions to ensure co-directionality of replication with transcription to minimize transcription-replication collisions. It would serve the field better if the authors can use hydroxyurea (HU) or other transient forms of nucleotide deprivation as another way to cause replication fork slow-down or fork stalling. Since this would affect all forks similarly (as opposed to APH, which would act stochastically), it would yield a more complete picture of whether genome-wide replication fork perturbations could lead to distinct CNAs or common CNAs in the two cell lines tested. HU can be used at low dose (200 microMolar) to ensure timely DNA replication while forks move slower requiring dormant origin firing, or can be transiently inhibited with high dose HU (2mM or higher at 4 hours or less) and wash off, to determine whether transient fork stalling could lead to any single cell cycle CNAs. Instead of using APH to get a somewhat unsurprising result, the authors, I believe, miss an opportunity to dive deeper in the effects of genome-wide replication stress. If the authors choose not to add additional data with HU, it would be important for them to explain how potential differences in replication stress inducers could alter the features of CNAs observed with APH. Too often, researchers use APH and HU interchangeably, which I think is mechanistically distinct in terms of fork perturbations.

**Authors Response**

**Point-by-point responses to the reviewers’ comments:**

**Reviewer 1:**

In this manuscript, authors used single cell sequencing to determine DNA copy number alterations (CNAs) that are induced by replication stress after one cell cycle. The study is performed in the absence of any selective pressure, which provides new information beyond previous study to detect CNAs that survive clonal outgrowth. However, some findings are surprising including very few recurrent CNAs at CFSs after aphidicolin treatment. This work would be potentially of interest to the field if major concerns are addressed.

1. It has been shown by different approaches that CFSs are the hot spots for chromosomal rearrangements and CNAs upon aphidicolin treatment. Hydroxyurea (HU) treatment causes breakage at early replicating fragile sites (ERFSs) that are distinctive from CFSs1. It will be informative to align all CNAs identified in this study with CFSs and ERFSs.

Response: *We thank the reviewer for this suggestion. We also initially found it interesting that so few sites were recurrently affected by large aphidicolin-induced CNAs, particularly in the RPE1 cells. However, we were recently able to compare these recurrent sites with RPE1-specific cytogenetically-mapped CFs provided by a recent study (Boteva et al, Cell Reports 20202). We also analysed proximity of BJ aCNAs with BJ-specific CFS mapped cytogenetically from another recent study (Sarni et al, Nature Communications, 20203). We have now included these locations in our diagrams of replication stress-induced CNAs (see Figures 2a and 3a) and also examined the proximity of our induced CNAs to those CFSs, as well as all CFSs identified from the literature (see Figure 4f). Interestingly we now see a significant correlation between recurrent large aCNA sites, and cell type-specific CFSs, highlighting the importance of considering cell type when analysing CFS. We also examined the relationship of our hydroxyurea-induced CNAs and indeed found a strong relationship with ERFS sites (see Figure 5e), a suggestion for which we also thank the reviewer.*

Authors claimed that "Different replicative stresses generated distinct profiles of CNAs". However, only aphidicolin treatment is shown in this study.

Response: *We thank the reviewer for pointing this out and note that reviewer three made a similar comment (see below). We have now included data after treating RPE1 cells with hydroxyurea, and compared the effects with those of aphidicolin (see Figures 1,2 and 5)*

2. It has been shown that DNA secondary structures are often hot spots for chromosomal breakage upon replication stress2. In this study, is there an overlap of mapped CNAs with DNA secondary structures?

Response: *We thank the reviewer for this helpful suggestion. We have used the secondary structure sequences predicted from the referenced publication (Shastri et al, Mol Cell, 20184) and looked for regions enriched for those sequences. We found that these sequences were interspersed throughout the human genome, but did find a mild correlation between the regions with highest (higher than median) concentrations of these repeats, and the probability of finding small losses generated by aphidicolin (p=0.04). We found no association with our list of random sites as a control, or sites where hydroxyurea treatments induced CNAs. We have now included this finding in the manuscript, in the section entitled “Repetitive regions prone to forming secondary structures associate with small loss aCNAs”.*

3. It is hard to find the concentration of aphidicolin used in this study for BJ and RPE cells. The concentration should be clearly indicated in the figure legends. It is important to show that single cell sequencing was carried out using the same concentration of aphidicolin and under the same condition that would induce CFS expression in BJ and RPE cells.

Response: *We apologise for not making this evident. We have made this clearer by explicitly stating the concentrations of aphidicolin as 0.4 µM (noted in the methods section as shown to induce CFS expression in RPE1 cells) and hydroxyurea as 2 mM, (see Figures 2a and 3a).*

4. It needs to show DNA replication timing and transcription profiles after aphidicolin treatment. This will provide additional information to understand the recurrent sites for CNAs in BJ and RPE cells.

Response: *We have now performed an additional single cell sequencing-based replication timing analysis for RPE1 cells treated with aphidicolin. We thus now include replication timing for both DMSO and aphidicolin-treated RPE1 cells in Figure 3f and Figure S6b,c. We noted that the trends were very similar between both replicating timing profiles, and that the associations between aCNAs and replication timing were also similar. We therefore also specifically analysed genomic regions where we saw the greatest shifts in replication timing after aphidicolin but did not note any significant associations except for large losses which were entirely accounted for by the recurrent aCNAs found at large gene AUTS2 (Figure S6d).*

*We did previously analyse gene expression in both DMSO, and aphidicolin conditions, however we have now made it more explicit about the extent to which transcription profiles are affected by aphidicolin in supplementary Figures S7a and S7c. We clarify that, overall, gene transcription is not changed for all genes, nor for all large genes, nor for the large genes we have focused on (AUTS2, MAGI2, NEGR1 and DAB1) between DMSO and aphidicolin treatments.*

5. MUS81 was shown to actively cleave underreplicated regions that are often present at CFSs3,4 to prevent chromosome bridging. It is surprising to see the features of CNAs in MUS81 knockdown cells are different from that in aphidicolin treated cells. To confirm the results, MUS81 knockout cells should be used. It is also important to show CNAs in MUS81 knockout cells after aphidicolin treatment, which would address whether the observed CNAs induced by aphidicolin are dependent on MUS81.

Response: *Indeed, in the publications you cited, the non-cancerous cell lines did show mild but significant increases in segregation errors from Mus81 depletion alone, which was then exacerbated by aphidicolin. In our hands, we did not observe differences between siCtrl and siMus81 in DMSO conditions in terms of visible segregation errors. This may be due to differences in cell lines; RPE1 cells may have a different level of reliance on Mus81 when compared to MRC5 cells or U2OS. Nonetheless, we did see elevated level of small CNAs not seen in DMSO treated cells (as shown in Figure 6b), although our ability to thoroughly analyse this was unfortunately confounded by the significant level of background noise (CNAs) in siCtrl DMSO, which became more evident after we sequenced additional cells for these experiments (see Figure S8b). Therefore, we have removed the analysis of features of siMus81 CNAs until we are able to build better cellular models that will allow us to address these more subtle phenotypes without the confounding effects of control siRNA treatment. Instead, we have now performed additional experiments and single cell sequencing to analyse the CNAs after siMus81 + aphidicolin. Despite the caveats relating to background CNAs (which we clearly explain in the manuscript), what we can clearly observe is that Mus81 depletion exacerbates the effect of aphidicolin, increasing the incidence of large terminal CNAs, which is broadly in line with the results of those papers (Figure 6f,g).*

*In terms of generating a Mus81 knockout cell line, we would argue against it at this time. The focus of this study was to examine the acute changes to the genome, immediately after one cell cycle of replication stress (or Mus81 depletion). Generating a knock-out cell line would require selection of cells with successful gene editing, which would take multiple cell doublings and rounds of the cell cycle to accomplish, during which the cells would accumulate a Mus81 k/o-derived repertoire of CNAs, which would then be shaped by selection processes. We hope that with the addition of single cell sequencing data in the condition of siMus81 combined with aphidicolin treatment, that now phenocopies results from prior studies, the siRNA-based data will be satisfactory for the purposes of this study.*

*Overall, we would like to thank you for your constructive comments which we feel have significantly strengthened our manuscript.*

**Reviewer 2**

In the manuscript "DNA Replication Stress Generates Distinctive Landscapes of DNA Copy Number Alterations and Chromosome Scale Losses"" Mazzagatti et al present data obtained from single cell sequencing that maps copy number alterations (CNAs) after one round of replication stress. They have used the non-transformed human cell lines RPE1-hTERT and BJ-hTERT. The authors analyse the CNAs according to replication timing and location of large and giant genes and complement their experiments with immunofluorescence assays.

Although single cell seq is an impressive technique this study does not significantly advances the understanding of replication-stress induced copy number alterations. Moreover, several claims are not adequately backed up by the data and finally citations and introduction to background literature are inaccurate.

Major points

1. Conclusions and interpretations are not adequately backed up

The conclusion that "large terminal CNAa but not small losses are associated with late replication timing" is surprising. It is clear from several published cancer genomic studies that small losses are associated with CFSs which in general have late replication timing. The authors should discuss why the method used here does not identify this well-established pattern.

In the figure text for fig 2 c, d and e it is important to write the total number of CNAs in each category. Is insufficient statistical power the reason that so few significant effects are observed throughout the paper?

Response: *We thank the reviewer for this comment, and have now added CNA numbers to figure legends (see Figure 2c and 3c). We have identified as many CNAs as we reasonably can, given the high cost of these experiments, by single-cell sequencing 168-332 cells. Using this data, and now including more analysis from the additional HU experiments, we have identified several major significant findings. First, aphidicolin induced CNAs are either small, or large, with large CNAs often originating at expressed large or giant genes, and in regions of late replication timing. Second, we identified an HU-specific signature of replication stress induced CNAs. Interestingly these CNAs do not behave in the same way as aphidicolin CNAs, and instead associate closely with ERFs. Therefore, although of course sequencing more cells would be desirable (and we are currently working to develop in-house single cell genomics which would allow this in future studies), we feel that we have been able to make significant progress, and identify several significant associations with the current numbers of cells sequenced.*

Fig 1L. It is surprising that APH has such small effect in particular in RPE1 cells where most categories are underrepresented after APH treatment compared to DMSO. It is highly problematic for any conclusions that relate to replication stress-induced CNAs.

Response: *When considering the rate of all CNAs per cell between DMSO and aphidicolin, we agree with the reviewer that only a small increase is evident (20 to 29 % cells with 1 or more CNAs; Figure 2b). However, this apparent small relative increase is due to a high background rate of CNAs (mostly small gains) in DMSO-treated cells. We attempted to ameliorate this effect, by discarding clonal events, or sub-cloning to generate a more homogeneous starting population, but were unable to do so. Much clearer increases in CNAs between DMSO and aphidicolin are evident when considering each CNA class separately, which we now present more clearly in Figure 2d in the form of a graph indicating the rate of each CNA class between DMSO, Aphidicolin, and HU treatments. Specifically, increases in small losses, and large losses and gains are seen in aphidicolin. HU also doubles the rate of small gains compared to DMSO. We agree that having larger increases would make it easier to determine features of replication stress induced CNAs, however for the purpose of this study we are limited to conditions that induce low levels of replication stress that allow cells to pass through mitosis and into G1.*

Fig. 3B.

What does the dots in gene expression actually represent? There is only on dot in AUTS2 -RPE cells. Does this mean that only one read was obtained?

Response: *We apologise that this was not clear in the original manuscript. Each dot in fact represents the mean (of three technical replicates) relative expression level of a single, expressed gene in the indicated cell line, at that location. Raw read counts are provided in Supplementary Table 3. We apologise that this was unclear and have clarified this in the figure legend (please note that this is now Figure 4d).*

When making any conclusions regarding expression of large and giant genes and their consequences for fragility it is very important to confirm that the entire gene is transcribed because the extreme size of the transcription unit is presumably the trigger for fragility. The authors should clearly indicate if they can confirm transcription throughout the large and giant genes, when they conclude whether or not transcription of large genes is a trigger for fragility. Also, it should be mentioned in the text if the depth used for RNAseq is sensitive enough to detect very low expression.

Response: *Our analysis used 3’ end RNAseq which is well suited to a quantitative analysis of gene expression precisely because it avoids bias from increased read counts from larger genes. We agree though, that it is possible that large gene expression signals could arise from shorter isoforms, rather than the full length transcripts (although this would not explain the very strong association between transcription of large/giant genes and fragility that we observed). To ask whether full length isoforms were expressed we performed an analysis (featureCounts, subread v2.0.1) to count reads at exon level using the GenCode annotation. From this analysis we assessed (by consulting the Ensembl database) whether there was evidence of transcripts from the exons expressed in the longest, or near-longest isoforms. Out of 79 large genes originally classed as expressed, we noted that whilst 75 appear to express the longest or near-longest isoforms, 5 only expressed a shorter isoform that covered the first few exons. We have corrected our analyses to take this into account by designating those as, essentially, non-expressing. Notably, the large genes discovered to be close to recurrent regions of aCNAs in RPE1 and BJ cells (AUTS2, MAGI2, DAB1, NEGR1) all have evidence of the longest isoform expression.*

*With regard to detection of very low gene expression, it is not easy to put a hard threshold on what depth of RNA sequencing is ideal to balance detection of lowly expressed genes with the cost of excess sequencing depth. In our analysis, ~13,000 genes were sufficiently detected and quantified which is quite routine for analyses of cell line monocultures. Therefore, we are likely picking up most genes that are expressed although we would have to perform a much higher read depth to fully determine the proportion of very lowly expressed genes that we did not detect in our analysis.*

For Fig. 4 I the n numbers are very small. Was the experiment only carried out once?

It should be carried out three times and then the authors must quantify a decent number from each experiment.

Response: (*There was no Figure 4l in the original submission but we think perhaps you are referring to original Figure 4b,c, or e). The original figures 4b and 4c relating to lagging chromosome centromere status and DNA damage were performed in triplicate and we have clarified this in the figure legend (note this is now Figure 1e and 1f). For original Figure 4e (micronuclei rates): DMSO treatment was originally only scored for one experiment, we have now performed additional experiments and now include this data in triplicate, whilst the aphidicolin was originally measured in triplicate. We have now also included hydroxyurea micronuclei data in triplicate and this is stated in the figure legend (note that this is now in Figure 1k). We apologise for any lack of clarity regarding this.*

In E it is not written what the error bars show.

Response: *In Figure 4e (now Figure 1k) the error bars were the standard deviation. We have clarified this in the figure legend and apologise for the omission.*

Fig. 5. The authors should explain why they do not see any signs of replication stress-induced aberration in mitosis after Mus81 depletion when looking at gH2AX, anaphase errors or UFBs. Yet the single cell seq suggest that there are mitotic aberrations with MUS81 knockdown. - Could these arise in S phase rather than by errors in mitosis?

Response: *The CNAs caused by Mus81 depletion alone in RPE1 cells were mostly focal changes which we identified through single cell sequencing but which could easily fail to be detected by microscopy. Indeed it is possible and a very interesting idea that these CNAs could be generated prior to mitosis during S-phase, hence not inducing visible mitotic DNA damage. This is a very interesting question that we are keen to address using our approaches to analyse cell cycle specific CNA induction and mechanistic origin in future studies.*

*Unfortunately, high rates of background CNAs in the control siRNA (which are more evident now that we have sequenced more siControl and siMus81 cells) makes it very difficult to clearly interpret these data. For this reason, we have removed the majority of analysis of siMus81 CNAs, apart from the analysis between aphidicolin, and siMus81+aphidicolin (Figure 6). In future, new cellular models to acutely remove specific replication stress pathway components will allow us to answer these questions more fully.*

In the discussion the authors state that "…. In RPE cells Mus81 is not essential for convertion of fragile sites into chromosome breaks …". The statement is based on the observation that Mus81 depletion did not reduce acentrentic lagging chromosomes and micronuclei. My feeling is that the authors have misunderstood the message in the cited paper (51), because the point of this paper is that microscopically visible "chromosome breaks", are in fact not DNA breaks but represent a repair intermediate. Consistently, in this paper it was already shown that MUS81 depletion leads to increased micronuclei formation. (In addition, the authors should also cite the back-to-back paper from Naim et al NCB 2013).

Response: *We have now cited the paper mentioned, and apologise for any confusion caused by our previous interpretations. We have amended our discussion of the effects of Mus81 to take into account the valid points made by the reviewer. Since we have now also added additional data comparing aphidicolin treatment with siMus81 + aphidicolin, and clearly see an increase in large terminal aCNAs in this latter condition, we can more clearly align our results with previous studies (please see section entitled “Depletion of Mus81 exacerbates the bias towards large aCNAs at recurrent sites in chromosomes 1, 2 and 7” on page 10, and Figure 6).*

2. The referencing is inadequate

Reference 1 and 15 is the same paper

Response: *We apologise for this mistake which was caused by an erroneous duplicate in our Endnote library, and have corrected this.*

In line 36-37, when mentioning oncogenes as drivers for replication stress why have the authors not cited the two hallmark papers here (Bartkova et al., 2005; Di Micco et al., 2006)?

Response: *We thank the reviewer for pointing this out and have now included these publications*

In line 42-44 MiDAS is introduced but the authors do not cite the first papers reporting DNA synthesis in mitosis (MiDAS) (Minocherhomji et al., 2015; Pedersen et al., 2015). It is correct that the term was coined in the (Bhowmick et al., 2016) paper, which is thus correctly cited.

Response: *We thank the reviewer and have now changed the references.*

In line 45 the authors write "…. Replication stress can induce lagging chromatin, anaphase bridges, aneuploidy and micronuclei formation". Again, the reference for this statement is insufficient. There are several papers reporting on these phenomena that came out before the cited paper - for instance (Chan et al., 2009; Ichijima et al., 2010; Naim and Rosselli, 2009)

Response: *We thank the reviewer for these suggestions and have corrected the citations.*

In line 53 the authors should also cite (Pentzold et al., 2018) for use of FANCD2 ChIPseq to map CFSs.

Response: *We have now included the paper in our citations.*

In the first result section 53BP1 NBs are analysed (Fig S1A). However, 53BP1 NBs have not been introduced and the authors have not cited the papers that first described 53BP1 here (Harrigan et al., 2011; Lukas et al., 2011).

Response: *We apologise for not citing the relevant paper. However, we have now decided to remove the 53BP1 data as it was not crucial, given that we already noted the effects of aphidicolin in inducing greater levels of acentric lagging chromosomes, micronuclei and RPA-coated ultrafine bridges.*

Minor points.

The second sentence in the abstract does not make sense

Response: *We have now corrected the sentence.*

Quantification of gH2AX foci in fig 1D and of 53BP1 in Fig S1 B and G and similar

quantifications should be represented by a super plot as explained in Lord et al, JCB,

20205

Response: *We thank the reviewer for this suggestion, and have altered those figures to be represented as super-plots in Figure 1 and supplementary Figures 2 and 5.*

It is not clear from figure or figure text what noc w/o refer to. It should be noted in the fig text or changed.

Response: *We apologise for not making this clear, we have clarified this in the main text, the methods and in the figure legend as referring to nocodazole washout.*

Line 41 p. Low levels of replication stress can bypass ATR (wrong to mention ATM here) as shown by Koundrioukoff et al.

Response: *We thank the reviewer for noting this error and have corrected it.*

**Reviewer 3:**

The manuscript Mazzagatti et al describes using a fairly novel technology of single cell sequencing to detect DNA copy number alterations (CNAs) to show how replication stress causes focal CNAs and chromosomal alterations (small and large scale gains or losses). The authors use two different diploid non-transformed cell lines for their analysis and carefully controlled for ensuring that the CNA analysis was caused by one cell cycle after undergoing replication stress. Although the results of the study was not unexpected (recurrent CNAs were largely confined to large genes that are somewhat transcribing), the potential of using this type of platform to study cancer genome evolution trajectories is intriguing. My only concern is the heavy reliance on low dose Aphidicolin (APH) for generating their single-cell CNA analysis. As it is well-understood that APH works to predominantly and irreversibly block Polalpha (lagging strand synthesis) activity, and also leading-strand, processive DNA polymerases (Pol delta/epsilon), this completely disrupts replication forks, and therefore requires the use of backup or dormant origins to fulfill DNA synthesis during S-phase in a timely manner. So low dose APH treatment is essentially a stochastic method of randomly blocking forks throughout the genome. So it is very obvious why these would be disproportionately affecting large genes in the human genome, as most replication forks (origin initiation) occurs at or near Transcription start sites of actively transcribing regions to ensure co-directionality of replication with transcription to minimize transcription-replication collisions. It would serve the field better if the authors can use hydroxyurea (HU) or other transient forms of nucleotide deprivation as another way to cause replication fork slow-down or fork stalling. Since this would affect all forks similarly (as opposed to APH, which would act stochastically), it would yield a more complete picture of whether genome-wide replication fork perturbations could lead to distinct CNAs or common CNAs in the two cell lines tested. HU can be used at low dose (200 microMolar) to ensure timely DNA replication while forks move slower requiring dormant origin firing, or can be transiently inhibited with high dose HU (2mM or higher at 4 hours or less) and wash off, to determine whether transient fork stalling could lead to any single cell cycle CNAs. Instead of using APH to get a somewhat unsurprising result, the authors, I believe, miss an opportunity to dive deeper in the effects of genome-wide replication stress. If the authors choose not to add additional data with HU, it would be important for them to explain how potential differences in replication stress inducers could alter the features of CNAs observed with APH. Too often, researchers use APH and HU interchangeably, which I think is mechanistically distinct in terms of fork perturbations.

Response: *We are pleased that the reviewer recognised the novel nature of the work and agree that utilising a second method of inducing replication stress would add further value to the conclusions. We tested a variety of doses of hydroxyurea, with both low, continuous doses (100-400 µM) or with a high dose (2 mM) for different timings of block and release (4 h, 12 h, 16 h block). We decided ultimately upon a 16 h block and release since that treatment schedule consistently generated increased micronuclei in the daughter cells (Figure 1). Whilst shorter time points might have led to CNAs independent of micronuclei formation (especially smaller CNAs), given the high costs of single cell sequencing we chose the option that generated micronuclei and more likely to generate CNAs detectable by both FISH and by sequencing. To our surprise, this HU treatment did not create a strong enrichment of specific chromosomes affected by CNAs (see Figures 1k, 2a, 2f) and in fact affected a much higher proportion of cells and generated a higher rate of CNAs than aphidicolin (see Figure 2b,c). We find that in fact there is little in common in the CNAs induced between the two treatments, and the genomic features we identified correlating with aphidicolin-induced CNAs had no such bearing on those triggered by hydroxyurea (Figure 5), which does indeed support your argument that they should not be used interchangeably. We therefore include this new data throughout the revised manuscript, mainly in Figure 1, and Figure 5, and would like to thank you for this important suggestion which we feel has significantly elevated the novelty and significance of our study.*

*1Barlow, J. H. et al. Identification of early replicating fragile sites that contribute to genome instability. Cell 152, 620-632, doi:10.1016/j.cell.2013.01.006 (2013).*

*2Boteva, L. et al. Common Fragile Sites Are Characterized by Faulty Condensin Loading after Replication Stress. Cell Rep 32, 108177, doi:10.1016/j.celrep.2020.108177 (2020).*

*3Sarni, D. et al. 3D genome organization contributes to genome instability at fragile sites. Nature communications 11, 3613, doi:10.1038/s41467-020-17448-2 (2020).*

*4Shastri, N. et al. Genome-wide Identification of Structure-Forming Repeats as Principal Sites of Fork Collapse upon ATR Inhibition. Molecular cell 72, 222-238 e211, doi:10.1016/j.molcel.2018.08.047 (2018).*

*5Lord, S. J., Velle, K. B., Mullins, R. D. & Fritz-Laylin, L. K. SuperPlots: Communicating reproducibility and variability in cell biology. The Journal of cell biology 219, doi:10.1083/jcb.202001064 (2020).*

**Second round of review**

**Reviewer 2**

In the revised manuscript “DNA Replication Stress Generates Distinctive Landscapes of DNA Copy Number Alterations and Chromosome Scale Losses” Shaikh et al present data obtained from single cell sequencing that maps copy number alterations (CNAs) after one round of replication stress caused by either APH or HU. They have used the non-transformed human cell lines RPE1-hTERT and BJ-hTERT. The authors analyse the CNAs according to replication timing and location of large and giant genes and complement their experiments with immunofluorescence assays and gene expression profiling.


This revised manuscript is a substantial improvement compared to the first version. The inclusion of experiments with HU treated cells and the careful comparison to results obtained with APH provides interesting new insight into the relation between replication stressors and chromosome fragility.

Here is just a few suggestions that would improve clarity

Page 2 line 57-58: Rephrase the sentence. As it stands now it gives the impression that gaps and breaks are named common fragile sites. It is more correct to write that the genomic regions susceptible to form gaps and breaks are named ”common fragile sites”

Page 10 line 317: change MiDas to MiDAS

Figure 1C. Use a black line around the big circles to make them more clear

Figure 2C and 3C: change aphid to Aph
